# NQO1 inhibits proteasome-mediated degradation of HIF-1α

**DOI:** 10.1038/ncomms13593

**Published:** 2016-12-14

**Authors:** Eun-Taex Oh, Jung-whan Kim, Joon Mee Kim, Soo Jung Kim, Jae-Seon Lee, Soon-Sun Hong, Justin Goodwin, Robin J. Ruthenborg, Myung Gu Jung, Hae-June Lee, Chul-Ho Lee, Eun Sung Park, Chulhee Kim, Heon Joo Park

**Affiliations:** 1 Department of Biomedical Sciences, College of Medicine, Inha University, Incheon 22212, Republic of Korea; 2 Hypoxia-related Disease Research Center, College of Medicine, Inha University, Incheon 22212, Republic of Korea; 3 Department of Biological Sciences, The University of Texas at Dallas, Richardson, Texas 75080, USA; 4 Department of Pathology, College of Medicine, Inha University, Incheon 400-712, Republic of Korea; 5 Division of Radiation Effects, Korea Institute of Radiological and Medical Sciences, Seoul 139-706, Republic of Korea; 6 Laboratory Animal Center, Korea Research Institute of Bioscience and Biotechnology, Yuseong-gu, Daejeon 305-806, Republic of Korea; 7 Department of Polymer Science and Engineering, Inha University, Incheon 22212, Republic of Korea; 8 Department of Microbiology, College of Medicine, Inha University, Incheon 22212, Republic of Korea

## Abstract

Overexpression of NQO1 is associated with poor prognosis in human cancers including breast, colon, cervix, lung and pancreas. Yet, the molecular mechanisms underlying the pro-tumorigenic capacities of NQO1 have not been fully elucidated. Here we show a previously undescribed function for NQO1 in stabilizing HIF-1α, a master transcription factor of oxygen homeostasis that has been implicated in the survival, proliferation and malignant progression of cancers. We demonstrate that NQO1 directly binds to the oxygen-dependent domain of HIF-1α and inhibits the proteasome-mediated degradation of HIF-1α by preventing PHDs from interacting with HIF-1α. NQO1 knockdown in human colorectal and breast cancer cell lines suppresses HIF-1 signalling and tumour growth. Consistent with this pro-tumorigenic function for NQO1, high NQO1 expression levels correlate with increased HIF-1α expression and poor colorectal cancer patient survival. These results collectively reveal a function of NQO1 in the oxygen-sensing mechanism that regulates HIF-1α stability in cancers.

## Abstract (web summary)

Elevated levels of the oxidoreductase NQO1 in tumours are associated with poor prognosis but how this contributes to cancer is poorly understood. Here, the authors find that NQO1 competes with PHD proteins, resulting in the stabilization of the hypoxia induced transcription factor HIF-1α.

NAD(P)H:quinone oxidoreductase 1 (NQO1, DT-diaphorase) is a cytosolic reductase which is upregulated in many human cancers^1^, including colorectal cancer^2^, lung cancer^3^, gastric cardiac carcinoma^4^, melanoma^5^, cholangiocarcinoma^5^, pancreatic cancer^5^, uterine cervical cancer^5^ and breast cancer^6^. In breast, colorectal and cervical cancers, the high-level expression of NQO1 is closely associated with the late clinical stage, poor differentiation and lymph node metastasis^5^,^6^. Consistently, breast and cervical cancer patients with high-level NQO1 expression have shown lower disease-free survival and 5-year overall survival rates compared with those with low-level NQO1 expression^5^,^6^. Despite the clear implications of NQO1 expression in the clinicopathological features and prognosis of these cancers, the molecular mechanisms underlying the pro-tumorigenic actions of NQO1 have not been fully elucidated.

Upregulation of NQO1 has been shown to protect cells against various cytotoxic quinones and oxidative stress; it catalyses the reduction and detoxification of quinone substrates, thereby protecting cells from diverse carcinogens^7^,^8^. Considerable efforts have been made to exploit the reductase activity of NQO1 to enhance the efficacy of certain bioreductive anticancer drugs^9^, such as, mitomycin C^10^, Geldanamycin^11^, E09^12^ and RH1^12^, 17AAG^13^. Hypoxia and high oxidative stress are among the hallmark features of the tumour microenvironment, yet the molecular effects of NQO1 on cell survival and bioreductive anticancer drugs within the hypoxic tumour microenvironment are largely unknown.

Hypoxia-inducible factors (HIFs) are critical transcription factors that regulate adaptive cellular responses to low O_2_ concentrations in metazoans^14^,^15^,^16^. HIFs have been reported to be overexpressed in various cancer cells under hypoxia commonly found in tumour microenvironments^16^,^17^. HIFs have been shown to regulate the expression of a number of genes involved in angiogenesis, tumour growth, metastasis, metabolic reprogramming, chemoresistance and radioresistance^16^,^17^,^18^.

HIFs are heterodimeric transcription factors that consist of three oxygen-regulated α subunits, HIF-1α, HIF-2α and HIF-3α, and a constitutively expressed hydrocarbon receptor/nuclear translocator β subunit, HIF-1β^19^,^20^,^21^. The HIF-1α and HIF-2α are structurally similar in their DNA binding and dimerization domains, but differ in their transactivation domains, thereby they have unique target genes^22^. HIF-3α is also similar in structure to the other two α-subunits but its function is less understood^21^. Formation of the HIF-1α and HIF-1β heterodimer is required for the function of HIF-1, wherein HIF-1α serves as the major regulatory subunit responsible for its transcriptional function^14^. The expression of HIF-1α is rapidly induced by hypoxia; when hypoxic cells are reoxygenated, the protein rapidly degrades (half-life of <10 min)^14^. In well-oxygenated cells, the oxygen-dependent degradation (ODD) domain of HIF-1α is hydroxylated by three prolyl hydroxylases (PHD 1–3), which utilize O_2_ and α-ketoglutarate as substrates^23^,^24^. The tumour suppressor, von Hippel-Lindau (pVHL) protein binds to hydroxylated HIF-1α and recruits an E3 ubiquitin ligase complex that includes Elongin-B, Elongin-C and Cullin2, thereby promoting ubiquitination and 26S proteasome-mediated degradation of HIF-1α^25^,^26^.

O_2_/PHD/VHL-independent mechanisms and other post-translational modifications have also been reported to be involved in regulating HIF-1α under normoxia and hypoxia^27^,^28^,^29^. For example, recent studies showed that the receptor of activated protein kinase C 1 (RACK1) competes with heat shock protein 90 (HSP90) for binding to HIF-1α and promotes the ubiquitination/degradation of HIF-1α by recruiting the E3 ubiquitin complex under normoxia^30^. Furthermore, HSP70 and CHIP (carboxyl terminus of the HSP70 interacting protein) promote the degradation of HIF-1α during prolonged hypoxia^31^. Cullin5, Bcl2 (B-cell lymphoma 2), Runx2 (Runt-related transcription factor 2) and factor inhibiting HIF-1 (FIH-1) are involved in the regulation of transactivation, stability and expression of HIF-1α^29^,^32^. Post-translational modifications of HIF-1α (for example, acetylation, phosphorylation, nitrosylation and SUMOylation) have also been reported, but their effects on the stability of HIF-1α remain controversial^27^,^28^,^29^.

In addition to its oxidoreductase activity, NQO1 has been shown to stabilize many proteins, including p53 and p33ING1b, by inhibiting their proteasomal degradation^33^,^34^. Here we report that NQO1 directly binds and stabilizes HIF-1α. We demonstrate that NQO1 binds to the ODD domain of HIF-1α and inhibits its polyubiquitination and proteasome-mediated degradation through competing with PHDs, regulatory proteins involved in its degradation. Consistently, high-level expression of NQO1 correlates with an increased expression of HIF-1α and poor survival in colorectal cancer patients. Collectively, this study reveals a role for NQO1 in the oxygen-sensing pathway that regulates HIF-1α stability in cancers.

## Results

### NQO1 increases poor prognosis in colorectal cancer

**Figure 1:**
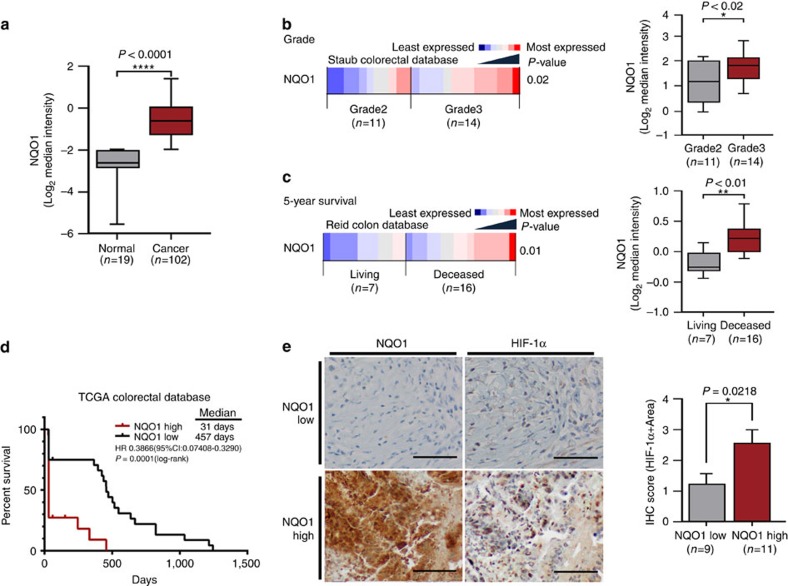
Upregulation of NQO1 correlates with poor prognosis and expression of HIF-1α in colorectal cancer. (**a**) An Oncomine analysis of the TCGA colorectal database indicated that NQO1 expressions are elevated in colorectal cancers (*n*=102) compared with normal colorectal tissues (*n*=19). ****P<0.0001 with unpaired *t*-test. (**b**,**c**) An Oncomine analysis of the Staub colorectal database (**b**) and the Reid database (**c**) indicate the elevated NQO1 mRNA expression correlates with the increased colorectal cancer grade (*n*=11 Grade 2, *n*=14 Grade 3; *P<0.02 with unpaired *t*-test) and that elevated NQO1 mRNA expression correlates with reduced patient survival at 5 years (**P<0.01 with unpaired *t*-test), respectively.The gene expression spectrum indicates that colours are *z*-score normalized to depict relative values within rows (left panel). They cannot be used to compare values between rows. For box plots, the centre line represents the median value, box limits are at the 25th and 75th percentiles, and whiskers represent minimum and maximum values (right panel). (**d**) NQO1 expression correlates with poor survival in colorectal cancer data set. Analysis of the colorectal cancer data set available through Oncomine indicates a significant correlation between the high-level expression of NQO1 and poor survival in the TCGA data set (*n*=21 NQO1 high, *n*=24 NQO1 low; *P*=0.0001 with log-rank analysis). HR, hazard ratio; CI, confidence interval. (**e**) Immunohistochemical detection of HIF-1α in the high-level expression of NQO1 (*n*=11) compared with the low-level expression of NQO1 (*n*=9). Positive area score of HIF-1α was determined on the most characteristic areas. The positive area score of HIF-1α was evaluated by determining 10 high magnification power fields ( × 40). Statistical analysis of the average score of HIF-1α positive area score is shown in the right panel (*P*=0.0218 with unpaired *t*-test). Scale bar, 100 μm.

Although NQO1 C609T polymorphism has been associated with increased colorectal cancer risk in recent meta-analysis studies^35^, the clinical significance of expression levels of NQO1 in human cancers has not been fully elucidated. Thus, we investigated the correlation between NQO1 expression levels and clinicopathological features in colorectal cancer patients utilizing publically available data sets. First, we found that the expression levels of NQO1 are significantly higher in colorectal cancer specimens compared with the matched normal colorectal specimens (Fig. 1a). Analysis of the Staub colorectal database^36^ indicates that the high levels of NQO1 expression significantly correlate with tumour grade (Fig. 1b). Analysis of the Reid colon database^37^ indicates that the high-level expression of NQO1 displays a negative prognostic value for long-term (>5 year) survival (Fig. 1c). To further evaluate the potential correlation of NQO1 expression with patient outcome, we performed a Kaplan–Meier survival analysis using TCGA (The Cancer Genome Atlas) colorectal cancer data sets revealing a strong correlation between high NQO1 expression and poor prognosis (Fig. 1d)^38^.

In an effort to identify mechanisms underlying the association of high NQO1 expression with increased malignance, we observed that hypoxic responses were dramatically attenuated in NQO1-knockdown cells. Thus, we sought to determine if HIF-1α expression correlates with NQO1 levels in human colorectal cancers. To validate specific upregulation of HIF-1α in NQO1-expressing tumours, we analysed HIF-1α protein levels in tumour tissues. Expression of HIF-1α, NQO1 or CA9 was defined by positive area score (Supplementary Fig. 1). Immunohistochemical analysis of colorectal cancer tissues demonstrates that high NQO1-expressing tumours, indicating 3 and 4 of IHC scores, contain a significantly elevated expression of HIF-1α (Fig. 1e). We further investigated the expression of known HIF-1α target genes by analysing TCGA RNA-seq and several publically available annotated colorectal cancer data sets in which we could compare the expression of NQO1 and HIF-1α target genes in colorectal cancer tissues with those of normal colorectal tissues. As illustrated in Supplementary Figs 2 and 3, most canonical HIF-1α target genes (CA9, LDHA, GLUT1, PDK1, PGK1 and vascular endothelial growth factor (VEGF)) were significantly elevated in high NQO1-expressing tumours as compared with those with absent or low NQO1 expression^38^,^39^,^40^,^41^, indicating that HIF-1α transcriptional signalling is accordingly activated in NQO1-expressing colorectal cancers. These results suggest that aberrantly elevated NQO1 expression correlates with the expression of HIF-1α and poor prognosis in colorectal cancer patients.

### NQO1 enhances HIF-1α expression

**Figure 2:**
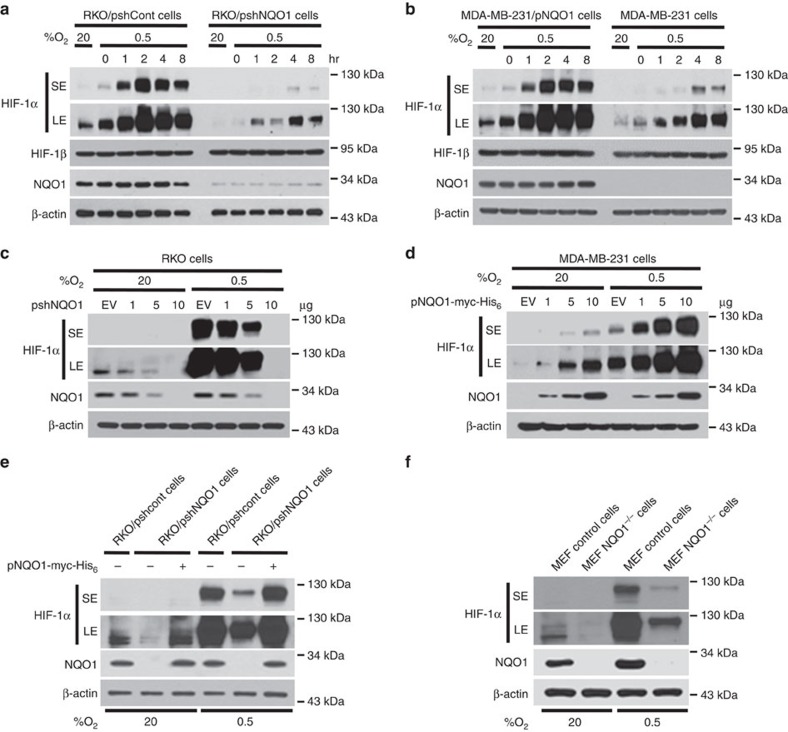
NQO1 enhances HIF-1α expression. (**a**,**b**) RKO cells (**a**) and MDA-MB-231 cells (**b**) were exposed to 0.5% O_2_ for 8 h and harvested at the indicated times. Whole-cell lysates were analysed by immunoblotting for HIF-1α, HIF-1β, NQO1 and β-actin. SE, short exposure; LE, long exposure. (**c**,**d**) Various concentrations of pshNQO1 and pNQO1-myc-His_6_ (0–10 μg) were transiently transfected into RKO (**c**) and MDA-MB-231 (**d**) cells, respectively. After 48 h, the cells were exposed to 20 or 0.5% O_2_ for another 2 h, and then harvested. Whole-cell lysates were analysed by immunoblotting for HIF-1α, NQO1 and β-actin. EV indicates pshCont (**c**) and pCDNA3.1-myc-His_6_ (**d**). SE, short exposure; LE, long exposure. (**e**) pNQO1-myc-His_6_ was transiently transfected into RKO/pshNQO1 cells. After 48 h, the cells were exposed to 0.5% O_2_ for 2 h, and then harvested. Whole-cell lysates were analysed by immunoblotting for HIF-1α, NQO1 and β-actin. SE, short exposure; LE, long exposure. (**f**) Mouse embryonic fibroblast wild type and NQO1^−/−^ cells were exposed to 20 or 0.5% O_2_ for 2 h and then harvested. Whole-cell lysates were analysed by immunoblotting for HIF-1α, NQO1 and β-actin. SE, short exposure; LE, long exposure.

To investigate the NQO1-mediated activation of HIF-1α signalling at the cellular level, we further assessed HIF-1α expression in different cell types with NQO1 gain-of as well as loss-of-function. First, we generated RKO human colorectal cancer cells that express short hairpin RNA (shRNA) targeting NQO1 (RKO/pshNQO1) and the control scramble shRNA (RKO/pshCont). When the RKO/pshNQO1 cells were exposed to hypoxia (0.5% O_2_), the expression of HIF-1α was markedly decreased as compared with control RKO/pshCont cells (Fig. 2a). Immunocytochemical staining consistently showed that NQO1 enhances HIF-1α expression under hypoxia (Supplementary Fig. 4a). We also exposed these cells to various oxygen tensions; 0.5, 1, 2, 5 or 20% O_2_, and we found that the knockdown of NQO1 significantly attenuates HIF-1α expression under mild hypoxia (Supplementary Fig. 4b). We next investigated the NQO1 gain-of-function in MDA-MB-231 human breast cancer cells that are deficient in NQO1 expression. As shown in Fig. 2b, overexpression of NQO1-myc-His_6_ (MDA-MB-231/pNQO1) dramatically increased the HIF-1α expression in both 20 and 0.5% O_2_. To further confirm that HIF-1α expression is regulated by NQO1, we transiently expressed various amount of pshNQO1 and pNQO1-myc-His_6_ in RKO and MDA-MB-231, respectively. Consistently, HIF-1α expression correlates with the levels of NQO1 expression (Fig. 2c,d). Ten human colorectal cancer cell lines and other types of cancer cell lines including HeLa (cervix), A549 (lung), PC3 (prostate) and U87-MG (brain) exhibited similar NQO1-mediated HIF-1α induction in both normoxia and hypoxia (Supplementary Fig. 4c,d). To exclude the possibility of off-site effects of NQO1 shRNA, NQO1 expression was restored by overexpressing shRNA-resistant NQO1 in RKO/pshNQO1 cells. As shown in Fig. 2e, reintroduction of NQO1 expression fully rescued the HIF-1α expression. In addition, immortalized mouse embryonic fibroblasts null for NQO1 (NQO1^−/−^) displayed markedly reduced levels of HIF-1α expression (Fig. 2f). We next sought to determine if NQO1 oxidoreductase enzymatic activity is necessary for HIF-1α induction. Introduction of point mutation at the known catalytic active site (C609T) that inactivates reductase activity had no effect on HIF-1α expression as compared with wild-type NQO1 indicating that NQO1 reductase enzymatic activity is not required for its HIF-1α inducing function (Supplementary Fig. 4e). Taken together, these results clearly suggest that NQO1 has a crucial role in HIF-1α expression independent of its reductase function.

### NQO1 increases HIF-1α stability

**Figure 3:**
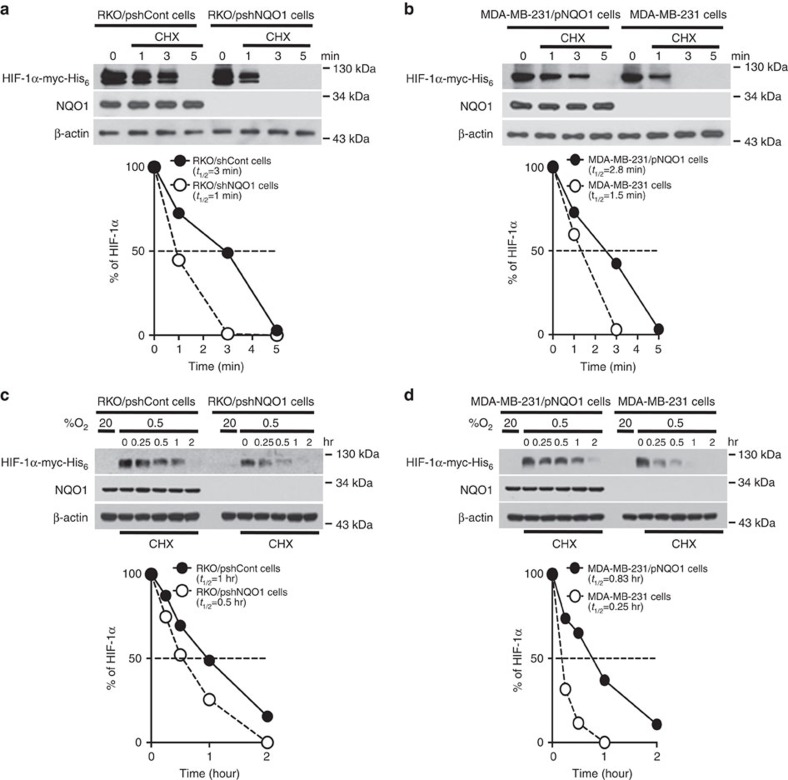
NQO1 regulates HIF-1α stability in cancer cells. (**a**,**b**) NQO1 increases the stability of HIF-1α protein under normoxia. Cells were transfected with pHIF-1α-myc-His_6_ and incubated with 10 μg ml^−1^ cycloheximide for 3 min, and then harvested at the indicated times. The protein levels of HIF-1α and NQO1 were examined by immunoblot analysis in RKO (**a**) and MDA-MB-231 (**b**) cells. β-actin was used as the internal control. (**c**,**d**) NQO1 prolongs the half-life of HIF-1α protein under hypoxia. Cells were transfected with pHIF-1α-myc-His_6_, exposed to 0.5% O_2_ for 2 h, incubated with 10 μg ml^−1^ cycloheximide for 2 h, and then harvested at the indicated times. The protein levels of HIF-1α and NQO1 were examined by immunoblot analysis in RKO (**c**) and MDA-MB-231 (**d**) cells. β-actin was used as the internal control. HIF-1α protein levels were quantified using Image J, and band intensities were normalized to those of β-actin (band intensity at *t*_0_ was defined as 100%).

To determine how NQO1 regulates HIF-1α expression, we first measured the messenger RNA (mRNA) expression levels of HIF-1α in NQO1 overexpressed or knockdown cells. *HIF-1α* mRNA expression was comparable among these cells independent of NQO1 levels regardless of O_2_ tension (Supplementary Fig. 5a). Additionally, we determined if NQO1 is involved in the regulation of *HIF-1α* mRNA stability. We treated cells with 5 μg ml^−1^ actinomycin D, which blocks *de novo* mRNA synthesis, under 20 or 0.5% O_2_ for 2 h. As shown in Supplementary Fig. 5b, the stabilization of the *HIF-1α* mRNA is not associated with NQO1 expression in the tested cells under both normoxia and hypoxia. These results suggest that *HIF-1α* mRNA transcription or degradation is not regulated by NQO1. Next, we sought to determine if NQO1 regulates HIF-1α protein stability. To investigate half-lives of HIF-1α, we introduced myc-His_6_ tagged HIF-1α (pHIF-1α-myc-His_6_) into NQO1 overexpressing or knockdown cells followed by treatment with 10 μg ml^−1^ cycloheximide, which blocks *de novo* protein synthesis under 20 and 0.5% O_2_. Under 20% O_2_, the half-lives of HIF-1α protein were decreased from 2.9±0.14 to 1.25±0.35 min as a consequence of NQO1 knockdown (RKO/pshNQO1) or NQO1 deficiency (MDA-MB-231), indicating that NQO1 stablizes HIF-1α protein (Fig. 3a,b). Consistently, under hypoxia, half-lives of HIF-1α protein were decreased from 1±0.12 to 0.5±1.41 h after cycloheximide treatment when NQO1 expression was suppressed (RKO/pshNQO1) or absent (MDA-MB-231) (Fig. 3c,d). These results suggest that NQO1 increases HIF-1α expression by regulating the stability of HIF-1α protein.

### NQO1 interacts with HIF-1α in the cytosol

**Figure 4:**
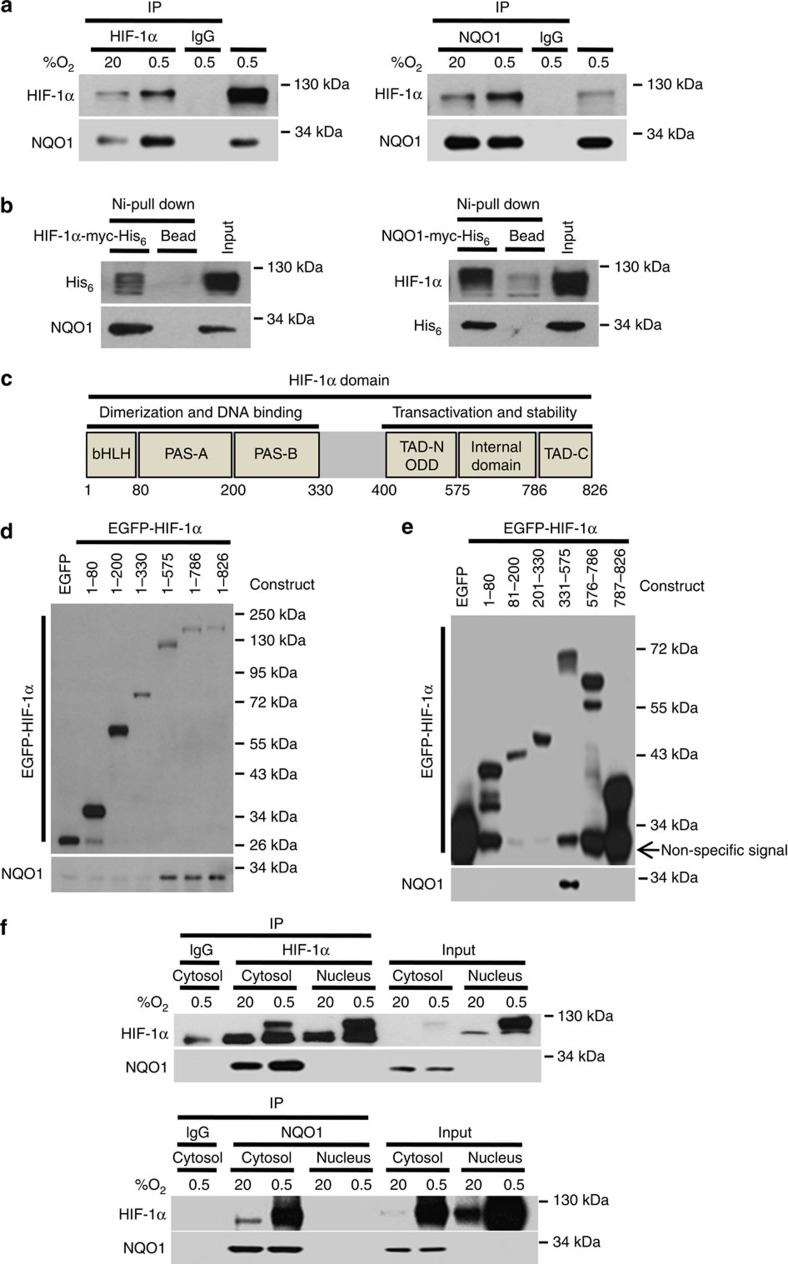
NQO1 interacts with HIF-1α in cytosol. (**a**) RKO cells were exposed to 20 or 0.5% O_2_. After 2 h, the cells were harvested and lysates were co-immunoprecipitated with anti-HIF-1α (left) or anti-NQO1 (right) antibody and anti-IgG antibody as a negative control. Then, the precipitates were analysed by immunoblot analysis with anti -HIF-1α and -NQO1 antibodies. (**b**) RKO cells were transfected with HIF-1α-myc-His_6_ (left) or NQO1-myc-His_6_ (right), subjected to Ni-NTA bead-based pulldown, and analysed by immunoblotting with anti-His_6_, -HIF-1α and -NQO1 antibodies. (**c**) Illustration of HIF-1α domain. (**d**,**e**) GFP pulldown assays were performed with purified EGFP fusion proteins containing the indicated amino-acid residues of HIF-1α, along with whole-cell lysates from RKO cells. The immunoprecipitated proteins were analysed by immunoblotting with anti-GFP and -NQO1 antibodies. (**f**) Extracted cytosolic or nuclear protein was immunoprecipitated with anti- HIF-1α (upper panel) or anti-NQO1 (lower panel) antibodies and analysed by immunoblotting to detect HIF-1α and NQO1.

We next assessed whether NQO1 is physically associated with HIF-1α as NQO1 has been shown to stabilize a number of proteins by physically binding to them^33^,^34^. The interaction between HIF-1α and NQO1 was determined by co-immunoprecipitation (Co-IP) as well as Ni-NTA bead-based pulldown assays. Co-IP results clearly indicated that NQO1 interacts with HIF-1α in RKO cells (Fig. 4a). Similar results were obtained from hypoxic mouse embryonic fibroblast cells (Supplementary Fig. 6). To determine if NQO1 directly interacts with HIF-1α, we employed Ni-NTA pulldown assays that suggest direct association of HIF-1α with NQO1 (Fig. 4b). To identify the binding motifs of the HIF-1α protein that interacts with NQO1 (Fig. 4c), we generated various HIF-1α deletion mutants linked to N-terminal EGFP fusion protein. As presented in Fig. 4d, NQO1 binds strongly to full-length and HIF-1α deletion mutants that included amino acid residues 1–575, 1–786 and 1–826 indicating that residues 331–575 of HIF-1α, which contain the ODD domain, are essential for the interaction with NQO1. To further pinpoint the potential-binding motifs, we generated additional HIF-1α deletion mutants that expand amino-acid residues 1–80, 81–200, 201–330, 331–575, 576–786 and 786–862 and subjected them to Co-IP assays for NQO1 binding. This revealed that NQO1 strongly binds to the ODD domain of HIF-1α (331–575) (Fig. 4e).

NQO1 is a cytosolic protein^1^, whereas HIF-1α translocalizes from cytosol into the nucleus once stabilized under hypoxia^29^,^42^,^43^. We thus performed Co-IP experiments in fractionated nuclear and cytosolic lysates to elucidate their cellular localizations of NQO1 and HIF-1α upon interaction. As indicated in Fig. 4f, NQO1 interacts with HIF-1α only in the cytosolic fractions. Altogether, these data provide evidence that NQO1 physically interacts with the ODD domain of HIF-1α proteins in the cytosol.

### NQO1 inhibits proteasome-mediated degradation of HIF-1α

**Figure 5:**
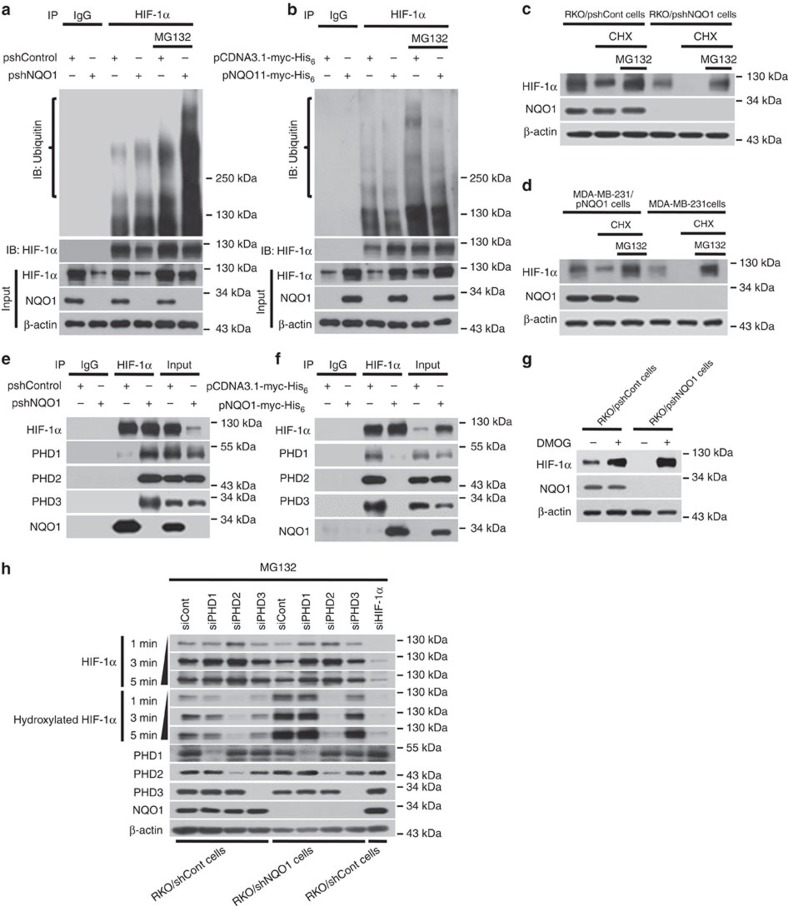
NQO1 stabilizes HIF-1α through inhibiting ubiquitination and proteasome-mediated degradation. (**a**,**b**) RKO (**a**) and MDA-MB-231 (**b**) cells were transfected with pshCont or pshNQO1 and pCDNA3.1-myc-His_6_ or pNQO1-myc-His_6_, respectively. The cells were then exposed to 0.5% O_2_ for 2 h, and incubated for 1 h in the presence or absence of MG132. Whole-cell extracts were immunoprecipitated with an anti- HIF-1α antibody, and ubiquitinated HIF-1α was detected with an anti-ubiquitin antibody. (**c**,**d**) RKO (**c**) and MDA-MB-231 (**d**) cells were exposed to 20 or 0.5% O_2_. After 2 h, the cells were treated with or without cycloheximide and MG132, incubated for 1 h, and harvested. The collected cells were analysed by immunoblotting for HIF-1α, NQO1 and β-actin. (**e**,**f**) RKO cells (**e**) and MDA-MB-231 cells (**f**) were exposed to 0.5% O_2_. After 2 h, the cells were harvested and immunoprecipitated with anti- HIF-1α or anti-IgG (negative control) antibodies. The precipitates were analysed by immunoblotting with the indicated antibodies. (**g**) RKO/pshCont and RKO/pshNQO1 cells were treated with or without 1 mM of DMOG for 1 h, and then exposed to 0.5% O_2_. After 2 h, the cells were harvested and analysed by immunoblotting with the indicated antibodies. (**h**) RKO/pshCont and RKO/pshNQO1 cells were transfected with siPHD1, siPHD2, siPHD3 or HIF-1α. After 48 h, the cells were treated with 50 μg MG132 for 6 h, exposed to 0.5% O_2_ for another 2 h, and then harvested. Whole-cell lysates were analysed by immunoblotting for HIF-1α, hydroxylated-HIF-1α, PHD1, PHD2, PHD3, NQO1 and β-actin. X-ray films were exposed for 1, 3 or 5 min to detect the signals of total HIF-1α, hydroxylated-HIF-1α.

Previous studies have shown that NQO1 stabilizes numerous proteins through inhibiting ubiquitination and the 26S proteasomal degradation^33^,^34^. To assess if NQO1 binding to the ODD domain of HIF-1α (Fig. 4e) accounts for the stabilization of HIF-1α via inhibiting ubiquitination and 26S proteasomal degradation, we measured the levels of ubiquitinated HIF-1α in cells with or without NQO1 expression. NQO1 knockdown (RKO/pshNQO1) and NQO1-deficient (MDA-MB-231) cells exhibited higher levels of ubiquitination in HIF-1α, whereas cells expressing high levels of NQO1 (RKO/pshCont and MDA-MB-231/pNQO1) suppressed ubiquitination in both hypoxia and normoxia (Fig. 5a,b and Supplementary Fig. 7a,b). To further investigate whether NQO1 inhibits HIF-1α degradation via the ubiquitin-mediated proteasomal pathway under hypoxia, we measured HIF-1α protein levels after treating cells with cycloheximide and/or MG132. As shown in Fig. 5c,d, MG132 treatment significantly inhibited the degradation of HIF-1α in NQO1-deficient cells. Collectively, these results indicate that NQO1 prevents HIF-1α from the ubiquitin-mediated proteasomal degradation.

Since the ODD domain of HIF-1α is hydroxylated by three PHD 1–3 in an oxygen tension-dependent manner that targets HIF-1α for ubiquitination and 26S proteasomal degradation^23^,^24^,^25^,^44^,^45^, we further investigated if the binding of NQO1 to the ODD domain of HIF-1α prevents PHDs from hydroxylating HIF-1α. First, Co-IP assays revealed that interaction between HIF-1α and PHDs was markedly reduced in NQO1-expressing RKO cells, but the interaction was fully rescued when NQO1 expression was suppressed by shRNA (Fig. 5e). Consistently, NQO1-deficient MDA-MB-231 cells exhibited an apparent interaction between PHDs and HIF-1α whereas, ectopic expression of NQO1 disrupted the interaction (Fig. 5f). To confirm whether PHDs participate in NQO1-mediated regulation of HIF-1α, we treated RKO/pshCont and RKO/pshNQO1 cells with dimethyloxloylglycine (DMOG), a potent PHDs inhibitor, under hypoxia for 2 h. As shown in Fig. 5g, inhibition of PHD activity by DMOG treatment abolished the reduction of HIF-1α expression caused by NQO1-deficiency clearly arguing for the functional involvement of PHDs in NQO1-mediated regulation of HIF-1α. To further confirm the functional contribution of PHDs, we transiently knocked down PHD1, PHD2 or PHD3 by siRNAs in control RKO and pshNQO1 RKO cells. As shown in Fig. 5h, HIF-1α expression levels in RKO/pshNQO1 cells remained comparable to control RKO cells when PHDs expression was suppressed. Accordingly, HIF-1α hydroxylation levels were significantly elevated in NQO1 knockdown RKO cells (RKO/pshNQO1), but knocking down PHDs effectively reduced HIF-1α hydroxylation and rescued HIF-1α expression. Although NQO1 appears to compete with all three PHDs to stabilize HIF-1α, consistent with previous studies, PHD2 is the predominant isoform involved in the hydroxylation of HIF-1α and NQO1 may stabilize HIF-1α mainly by blocking PHD2 binding to the ODD domain. We further employed ODD-luciferase reporter system to validate the functional contribution of NQO1 to inhibition of PHD-mediated hydroxylation and degradation of HIF-1α. Even under hypoxia, NQO1 knockdown significantly induced the degradation of HIF-1α, which was partially rescued by siRNA-mediated suppression of PHDs (Supplementary Fig. 7c). Altogether, these results provide evidence that NQO1 stabilizes HIF-1α through competing with PHDs for binding to the ODD domain of HIF-1α.

### NQO1 increases HIF-1α transcriptional activities

**Figure 6:**
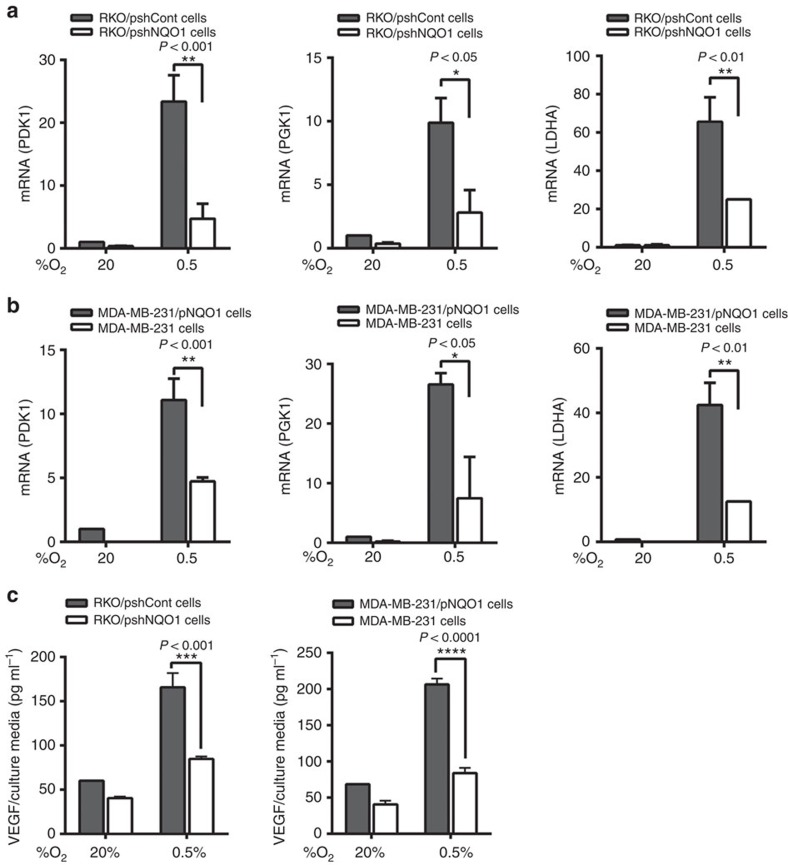
**NQO1** **increases transcriptional activity of HIF-1α in cancer cells under hypoxia.** (**a**,**b**) RKO (**a**) and MDA-MB-231 (**b**) cells were transfected with pshCont or pshNQO1 and pCDNA3.1-myc-His_6_ or pNQO1-myc-His_6_, respectively, exposed to 20 or 0.5% O_2_ for 8 h, and then harvested. Quantitative PCR was used to amplify PDK1, PGK1 and LDHA. The signals were normalized by *18S rRNA*. (mean±s.d. shown) *n*=3 in each group. *P<0.05 with analysis of variance (ANOVA), **P<0.01 with ANOVA. (**c**) RKO (left panel) and MDA-MB-231 (right panel) cells were transfected with pshCont or pshNQO1 and pCDNA3.1-myc-His_6_ or pNQO1-myc-His_6_, respectively, exposed to 20 or 0.5% O_2_ for 24 h, and then the conditioned media was harvested. Enzyme-linked immunosorbent assay was used to analyse the secretion of VEGF in conditioned media. (mean±s.d. shown). *n*=3 in each group. ***P<0.001 with ANOVA, ****P<0.0001 with ANOVA.

We next assessed if NQO1-mediated HIF-1α stabilization functionally leads to transcriptional activation of its target genes. Under hypoxia, knocking down NQO1 in RKO cells (RKO/pshNQO1) dramatically attenuated hypoxic induction of HIF-1α target genes, PDK1, PGK1 and LDHA (Fig. 6a). Moreover, ectopic expression of NQO1 in NQO1-deficient MDA-MB-231 cells further increased the expression of these target genes (Fig. 6b). To confirm this transcriptional activity, we transfected cells with a hypoxia reporter plasmid (p5 × HRE-*luc*) and a transcription control, pCMV-β-galactosidase. We found that, under hypoxia, HRE transcriptional activities were significantly suppressed when NQO1 expression was reduced by shRNA (RKO/pshNQO1) whereas NQO1-overexpressing cells exhibited elevated HRE-luc activities compared with NQO1-deficient MDA-MB-231 cells (Supplementary Fig. 8a). Lastly, we measured another HIF-1α target gene, VEGF in the conditioned media. Enzyme-linked immunosorbent assay assays showed a decreased concentration of secreted VEGF from NQO1-knockdown RKO cells and increased levels of VEGF from NQO1-overexpressing MDA-MB-231 cells (Fig. 6c), which was consistently associated with angiogenic activities evaluated by *in vitro* tube formation capacities of human umbilical vein endothelial cells (Supplementary Fig. 8b). Taken together, these results indicate that NQO1-mediated HIF-1α expression promotes HIF-1α transcriptional activities and NQO1 inhibition significantly attenuates target gene expression, suggesting that NQO1 is a critical component for full transcriptional activation of the HIF-1α signalling pathway under hypoxia.

### NQO1 promotes *in vivo* tumour growth

**Figure 7:**
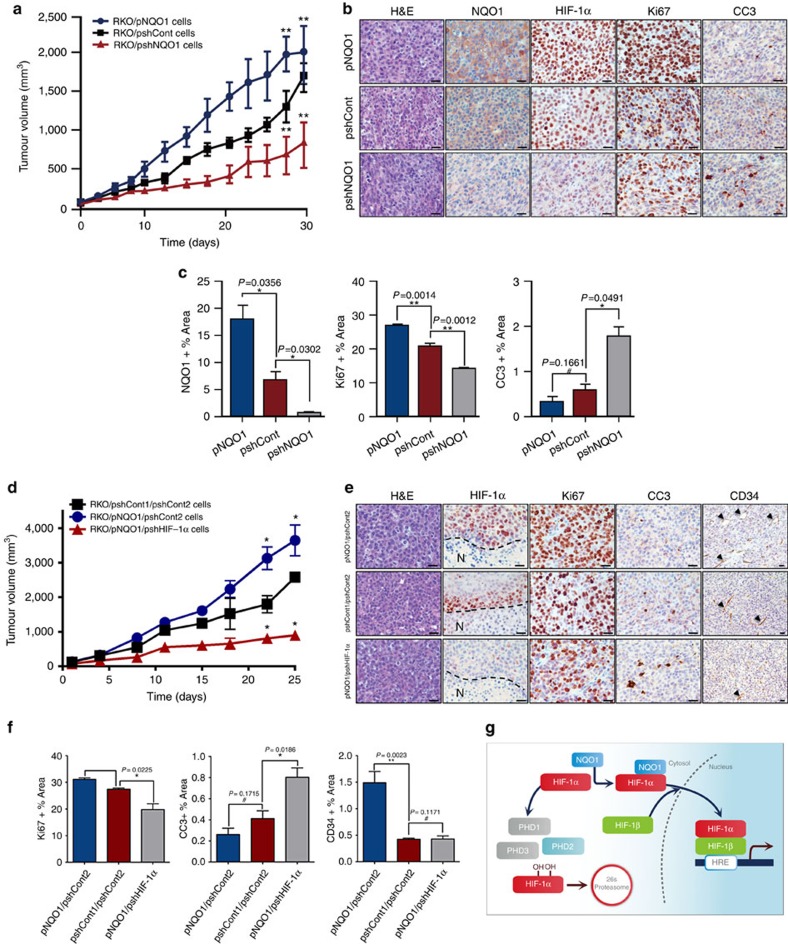
**NQO1 promotes** ***in vivo***** tumour growth.** (**a**) RKO/pNQO1, RKO/shCont and RKO/pshNQO1 cells were injected subcutaneously into the right flank of athymic, 7-week-old female BALB/C nude mice, and tumour growth was assessed. Tumour volume (TV) was calculated by using the following formula: TV=length × (width)^2^ × 0.5. Each group contained 12 animals. (**P<0.01 with unpaired *t*-test). (**b**) Immunohistochemical analyses of RKO/pNQO1, RKO/shCont, and RKO/pshNQO1 xenograft tumours. The sections were stained for NQO1, HIF-1α, proliferation (Ki67) and apoptosis (CC3) using 3,3′-DAB. Scale bar, 50 μm. (**c**) Quantification of NQO1, proliferative marker Ki67 and apoptotic marker CC3 in RKO/pNQO1, RKO/shCont and RKO/pshNQO1 xenograft tumours (*n*=3 each group). *n*=5 in each tumour. Two-tail *t*-test. **P<0.01, *P<0.05. #, not significant. All error bars represent the mean±s.e.m. (**d**) RKO/pshCont1/pshCont2, RKO/pNQO1/pshCont2 and RKO/pNQO1/pshHIF-1α injected subcutaneously into the right flank of athymic, 7-week-old female BALB/C nude mice, and tumour growth was assessed. Tumour volume (TV) was calculated by using the following formula: TV=length × (width)^2^ × 0.5. Each group contained 10 animals (*P<0.05 with unpaired *t*-test). (**e**) Immunohistochemical analyses of RKO/pshCont1/pshCont2, RKO/pNQO1/pshCont2 and RKO/pNQO1/pshHIF-1α xenograft tumours for HIF-1α, proliferation (Ki67), apoptosis (CC3) and vasculature (CD34) using 3,3′-DAB. Arrowheads denote blood vessels. Scale bar, 50 μm. (**f**) RKO/pNQO1/pshCont or RKO/pNQO1/pshHIF-1α xenograft tumours (*n*=3–5 each group) were quantified for proliferation (Ki67), apoptosis (CC3) and vascularization (CD34). *n*=5 in each tumour. Two tailed *t*-test. **P<0.01, *P<0.05. #, not significant. All error bars represent the mean±s.e.m. (**g**) Schematic model showing how NQO1 stabilizes HIF-1α in cancer cells.

To determine the functional contribution of NQO1 to HIF-1α expression and tumour growth *in vivo*, we evaluated the growth rate of NQO1-knockdown or overexpressing RKO xenografts in female BALB/c nude mice. As illustrated in Fig. 7a, NQO1 knockdown tumours displayed dramatically reduced growth rate, whereas tumours overexpressing NQO1 grew much faster as compared with control tumours (Fig. 7a). We assessed the levels of NQO1 and HIF-1α in these tumours and found that NQO1 knockdown tumours showed significantly reduced HIF-1α expression, which was associated with decreased proliferation (Ki67) and elevated apoptosis (cleaved caspase 3, CC3). In sharp contrast, NQO1 overexpressing tumours showed accelerated growth rate with higher cellular proliferation (Fig. 7b,c). We further assessed HIF-1α expression in relation to hypoxic areas by co-staining with hypoxia marker, pimonidazole, and HIF-1α. Control RKO tumours exhibited clear correlative HIF-1α expression along hypoxic areas. However, HIF-1α-positive areas were substantially expanded over the pimonidazole-positive hypoxic areas in NQO1-overexpressing tumours (RKO/pNQO1). Conversely, NQO1-knockdown tumours (RKO/pshNQO1) showed significantly less HIF-1α expression even in the pimonidazole-positive hypoxic areas (Supplementary Fig. 9b). To confirm that this reduction in tumour growth is specifically due to a lack of HIF-1α stabilization by NQO1, rather than through other NQO1 mediated effects, we studied the growth rate of control (RKO/pshCont1/pshCont2), NQO1 overexpressing (RKO/pNQO1/pshCont2), and NQO1 overexpressing but HIF-1α knockdown (RKO/pNQO1/pshHIF-1α) xenograft tumours. Consistently, HIF-1α-knockdown almost completely inhibited tumour growth, even in the presence of overexpressed NQO1 (Fig. 7d). This suggests that tumour growth is inhibited specifically through reduced NQO1-dependent HIF-1α expression. We further validated these results by quantifying Ki67 and CC3 expression and found that in accordance with our previous results, decreased HIF-1α expression in RKO tumours was associated with decreased proliferation (Ki67) and elevated apoptosis (CC3) (Fig. 7e,f). Intriguingly, we found that NQO1 overexpression enhanced microvessel density while there was no difference in vascular density between control and HIF-1α knockdown tumours, measured using CD34 staining (Fig. 7e,f). Collectively, these observations strongly argue for the functional contribution of NQO1 in stabilizing HIF-1α which, in turn, enhances tumour growth *in vivo*.

## Discussion

The present study demonstrated for the first time that NQO1 directly binds to HIF-1, thereby it stabilizes HIF-1 protein and enhances the tumorigenic activity of HIF-1α. NQO1 is a ubiquitously expressed FAD (flavin adenine dinucleotide)-dependent flavoprotein that provides essential cytoprotective anti-oxidant functions through catalysing a two-electron reduction of potentially toxic quinones^7^,^8^. NQO1 polymorphisms, particularly C609T that causes rapid ubiquitination and degradation of the NQO1 protein, have been implicated with a higher risk of several human cancers^46^,^47^, but, paradoxically, upregulation of NQO1 has been also shown to correlate with poor prognosis of colorectal, breast and cervical cancers^5^,^6^. Here we revealed previously unsuspected pro-tumorigenic roles for NQO1 that essentially increase the expression of the HIF-1α protein, a central transcriptional factor of the hypoxic response of cancer cells to the hypoxic tumour microenvironment. We further provide evidence suggesting that NQO1 stabilizes the HIF-1α protein by physically interacting with the ODD domain of HIF-1α that prevents PHDs from binding to HIF-1α. Given that HIF-1α has a prominent role in hypoxic adaptation and tumorigenic progression in most human cancers^16^,^17^,^18^, our study provides a possible mechanistic explanation for the involvement of NQO1 overexpression with poor prognosis in clinical outcomes of cancer patients.

In order to better understand the potential clinical implication of NQO1 for the prognosis of human cancer, we revisited publically available data sets for colorectal cancer. As reported previously by others^38^, the NQO1 level in colorectal cancer specimens was significantly higher than that in NQO1 expression in the normal colorectal tissue. We also observed that the cancer tissues expressing high NQO1 level also express higher-level of HIF-1α protein than tumours expressing relatively low NQO1 (Fig. 1e). As shown in Fig. 2, we identified a function of NQO1 in which HIF-1α stability was markedly enhanced under hypoxia following ectopic introduction of NQO1 into NQO1-deficient MDA-MB-231 breast cancer cells. Conversely, RKO colorectal cancer cells with suppressed NQO1 expression showed markedly accelerated degradation of HIF-1α. We further observed that HIF-1α mRNA transcription is independent of NQO1 (Suppelmentary Fig. 5), and that the degradation of HIF-1α protein is prevented by NQO1 via direct physical interaction between NQO1 and HIF-1α proteins in the cytosol (Fig. 4). In this context, it has been reported that NQO1 stabilized several proteins including CEBPα, p53, p73 and p33ING1b by blocking their proteasomal degradation^33^,^34^.

Oxygen-dependent hydroxylation of HIF-1α by PHDs has been a well-acknowledged central oxygen-sensing pathway that regulates the HIF-1α protein stability. Our findings that NQO1 directly forms a complex with the ODD domain of HIF-1α competing with PHDs for binding, thereby protecting HIF-1α from ubiquitin-mediated proteasomal degradation (Fig. 5) add another layer of regulation to the cellular oxygen homeostasis. In a previous report, PHD2 was the key regulator setting low steady-state levels of HIF-1α^48^. In accord with this report, we also observed that low expression of NQO1 decreased steady-state levels of HIF-1α (Fig. 2), and that inhibition of PHD2 by siRNA decreased hydroxylation of HIF-1α in NQO1-deficient cell lines (Fig. 5h). Yet, our study cannot entirely rule out the possibility that NQO1 may enhance the HIF-1α expression also through regulating the degradation of other proteins that affects the HIF-1α protein stability. For instance, recent studies have demonstrated that HSP90 increased the stability of HIF-1α proteins, whereas HSP70, Cullin5 and RACK1 decreased the stability of HIF-1α protein^30^,^31^,^32^. Moreover, a previous study has shown that HIF-1α is degraded by HSP70 via a PHDs-independent mechanism during prolonged hypoxia^31^. However, expression of these proteins was not affected by NQO1 (Supplementary Fig. 10a,b).

Although HIF-1 signalling has been associated with increased mortality of most human cancers through multiple aspects of the pro-tumorigenic process including angiogenesis, tumour growth, metastasis, chemoresistance and radioresistance^16^,^17^,^18^, recent studies reported that HIF-1α could act as a tumour suppressor in certain cancers such as VHL-null renal cell carcinoma where HIF-2α functions as a dominant oncogenic driver. Moreover, increasing evidence suggests that functional contributions of HIF-1α and HIF-2α to tumour progression are context dependent. Indeed, we also observed enhanced expression of HIF-2α by NQO1 under hypoxia (Supplementary Fig. 11a,b). Thus, it would be a great importance to warrant further investigation to better understanding of the roles of NQO1 in the context of complex relationship between HIF-1α and HIF-2α.

Despite the fact that NQO1 has been reported to be highly expressed in numerous human cancers and our tumour xenograft experiments clearly argue that NQO1 is pro-tumorigenic (Fig. 7a), contradictory results on its biological roles in tumorigenesis have been reported. As mentioned above, previous studies have demonstrated that NQO1 plays a critical role in cellular defense mechanisms against free radical damage, oxidative stress, inflammatory responses and carcinogenesis^33^,^49^,^50^. NQO1-null mice exhibited increased susceptibility to chemical-induced skin carcinogenesis^51^. Moreover, NQO1 has been shown to stabilize tumour suppressor, p53 (ref. 33). In addition, it has been reported that NQO1 suppresses NF-κB-p300 interaction to regulate inflammatory mediators associated with prostate tumorigenesis^52^. These studies suggested that NQO1 could act to suppress tumorigenesis in certain conditions. Although these anti-tumorigenic effects of NQO1 appear to be due to protective functions of its oxidoreductase activities, non-catalytic roles, such as stabilization of p53, may be involved in its tumour suppressive roles. More studies will be required to explore a dual function of NQO1 in different cancers and more importantly, how HIFs contribute to this dual function of NQO1 in tumorigenic progression. Previously, Dang *et al*.^51^ demonstrated that HIF-1α knockout had no effect on the growth of RKO xenografts. While this is inconsistent with our observations that HIF-1α knockdown attenuated RKO xenograft growth compared with control and NQO1 overexpressed tumours, histological evaluation of these tumours showed reduced proliferation and increased apoptosis in HIF-1α-deficient xenografts. These results agree with observations of reduced tumour burden upon HIF-1α knockdown or knockout in several other colon cancer cell lines, though further studies are needed to determine possible compensatory mechanisms upon complete HIF-1α knockout and whether they are specific to RKO cells or other colon cancer cell lines. Consistent with Dang *et al*.^51^ we observed no difference in microvessel density upon HIF-1α knockdown. However, NQO1 overexpression displayed increased vasculature compatible with our observations of enhanced VEGF production *in vitro* upon NQO1 overexpression.

In summary, our findings indicate that NQO1 enhances HIF-1 activity by stabilizing the HIF-1α protein (Fig. 7g). Remarkably, hypoxic accumulation of HIF-1α was further amplified in NQO1 expressing cancer cells that may accelerate the tumour growth. Our insight into PHDs-mediated oxygen sensing machinery in cancer leads to a discovery of a regulator of HIF-1α stabilization that may open up an avenue for anti-cancer therapeutic strategies.

## Methods

### Cell lines and culture conditions

A549 human lung adenocarcinoma epithelial cells, HeLa human cervical cancer cells, PC3 human prostate cancer cells, MDA-MB-231 human breast cancer cells and U87MG glioblastoma cells were obtained from American Type Culture Collection and cultured in Dulbecco's modified Eagle's medium (DMEM) or RPMI medium. Human colorectal cancer cell lines, including RKO, HT-29, SW403, LST174T, CoLo205, HCT15, SW116, SW948, SW620, SW480 and DLD-1, were obtained from American Type Culture Collection and cultured in DMEM or RPMI medium. Cells were incubated at 37 °C in a 5% CO_2_-containing humidified incubator unless otherwise noted. Human umbilical vein endothelial cells were obtained from Sciencell Research Laboratories. The cells were cultured in complete endothelial cell medium (Sciencell Research Laboratories) supplemented with 5% fetal bovine serum, 1% antibiotics and 1% endothelial cell growth supplement. Cells from passages 2∼5 were used for experiments. Wild-type and NQO1(^−/−^) cells were kindly provided by Dr Lee, C.H. (Laboratory Animal Resource Center, Korea Research Institute of Bioscience and Biotechnology, Daejeon, Republic of Korea). Cells were maintained in DMEM supplemented with 10% fetal bovine serum, 1% antibiotics at 37 °C in a 5% CO_2_-containing humidified incubator. Cells from passages 3∼4 were used for experiments. For hypoxic exposures, appropriated numbers of cells in 5 ml of medium were seeded in each specially designed 25-cm^2^ glass flasks coated with 0.01% gelatin and incubated overnight under standard culture conditions. The flasks were then sealed with rubber stoppers and two stainless steel needles (18-gauge) were pierced through each rubber stopper to provide entrance and exit ports for gas. Stoppered glass flasks were gassed with a humidified mixture of 5% CO_2_ and 95% N_2_ gas for 1 h at 37 °C through one of the needles. All cell lines have been tested for the presence of mycoplasma by PCR.

### NQO1 gene expression in human colorectal cancers

Correlations between tumour grade, patient survival, NQO1 and CA9 gene expression were determined through analysis of TCGA, Ki, Skrzypczak, Kaiser, Staub, Reid and Smith data sets, which are available through Oncomine (Compedia Biosciences, http://www.oncomine.org/). High and low groups were defined as above and below the mean value, respectively.

### Patients and specimens

Written informed consent approved by the Institutional Review Board of Inha University Hospital Clinical Trial Center (IRB No. 14-052; Incheon, Korea) was obtained from all the patients who were to undergo colorectal resection for colorectal cancer. Surgically resected colorectal cancer tissues were immediately fixed in 10% neutral formalin for 24–72 h. Haematoxylin-eosin stained slides were made according to the routine procedure and microscopic examination was done by a pathologist. Twenty cases of adenocarcinoma were randomly selected from the pathologic file in Inha University Hospital in November 2013. Pathological diagnosis, histological type, tumour stage and tumour percentages were determined.

### Chemicals and antibodies

Actinymycin D, cycloheximide and MG132 were purchased from Calbiochem (Merck KGaA). DMOG was purchased from Santa Cruz Biotechnology. Antibodies were obtained from the following sources: Anti-HIF-1α (1:500; MAB1536, R&D Systems), anti-PHD1 (1:1,000; AF6394, R&D Systems), anti-Hydroxylated-HIF-1α (1:1,000; #5853S, Cell Signaling Technology), anti- HIF-1β (1:1,000; #3414S, Cell Signaling Technology), anti-PHD2 (1:1,000; #3293, Cell Signaling Technology), anti-RACK1 (1:1,000; #5432S, Cell Signaling Technology), anti-Ki67 (1:200; #12202, Cell Signaling Technology), anti-CC3 (1:200; #9664, Cell Signaling Technology), anti-PHD3 (1:1,000; NB-100-139, NOVUS), anti-NQO1 (1:2,000; 39–3,700, Invitrogen), anti-ubiquitin (1:500; 131,600, Invitrogen), anti-β-actin (1:5,000; A5316, Sigma-Aldrich), anti-Cullin5 (1:1,000; sc-13014, Santa Cruz Biotechnology), anti-HSP70 (1:2,000; ADI-SPA-820D, Stratagene), anti-HSP90 (1:2,000; ADI-SPA-830D, Stratagene), anti-His_6_ (1:1,000; 11 911 416 001, Roche Applied Science), anti- GFP (1:1,000; 11 814 460 001, Roche Applied Science), anti-CD34 (1:200; ab8158, Abcam), anti-pimonidazole (1:200; HP6-x, Hydroxyprobe). The secondary antibodies were obtained from the following sources: Anti-mouse-fluorescein isothiocyanate (FITC; 1:200, sc2010, Santa Cruz Biotechnology), anti-rabbit-TR (1:200; A-11011, ThermoFisher Scientific), anti-mouse-HRP (1:2,000; #7076S, Cell Signaling Technology), anti-rabbit-HRP (1:2,000; #7074S, Cell Signaling Technology). The antibodies used as negative controls for immunoprecipitation included normal mouse IgG (sc-2025, Santa Cruz Biotechnology) and normal rabbit IgG (sc-2027, Santa Cruz Biotechnology).

### Construction of plasmids and stable cell lines

To construct the plasmid encoding five tandem copies of an HRE, the *VEGF* regulatory region was constructed by ligating two oligonucleotide ligands (5′-TCG AGC CAC AGT GCA TAC GTG GGC TCC AAC AGG TCC TCT TG-3′ and 5′-TCG ACA AGA GGA CCT GTT GGA GCC CAC GTA TGC ACT GTG GC-3′), and subcloning this fragment into pGL3-Basic (Promega) upstream of the gene encoding firefly luciferase. To construct the plasmids expressing, NQO1, HIF-1α and the EGFP-HIF-1α fusion protein, total RNA was obtained from RKO cells using the TRIzol reagent (Invitrogen) and complementary DNA (cDNA) was generated using SuperScriptIII Reverse Transcriptase (Invitrogen). The open reading frames (ORFs) of NQO1 and HIF-1α were PCR (polymerase chain reaction) amplified with appropriate primers. The PCR products were digested with restriction enzymes and directly ligated into the pCDNA3.1-myc-His_6_ (Invitrogen), pCDNA-EGFP (Addgene) or pEGFP-c1 (Clonetech) vectors for cloning. The cloned plasmids were analysed by restriction digestion and DNA sequencing (Bionics, Seoul, Republic of Korea). pODD-luc were purchased from Addgene (Cambridge, MA, USA). To construct stable cell lines, cells were seeded at 5 × 10^4^ cells per well in 24-well plates and transfected with 50 μl of a mixture containing 1 μg of pCDNA3.1-NQO1-myc-His_6_, pshNQO1 or pshHIF-1α (Qiagen), along with the TurboFect *in vitro* transfection reagent (Fermentas). As controls, we used pCDNA3.1-myc-His_6_ and pshCont (Qiagen). Transfected cells were selected with 1 mg ml^−1^ G418 or 1 μg ml^−1^ puromycin (DuchefaBiochemie, Haarlem, Netherlands) for 1 week and maintained in DMEM or RPMI-1640 containing 0.5 mg ml^−1^ G418 or 0.3 μg ml^−1^ puromycin during the experiments.

### Transfection assays

Cells (5 × 10^4^) were seeded into glass tubes with 2-cm^2^ flat walls (Bellco Glass; Catalog number#1908-16125) coated with 0.01% gelatin, incubated overnight, and co-transfected with 50 μl of a mixture containing 1 μg of pODD-*luc* or p5 × HRE-*luc*, 0.1 μg of pCMV-β-galactosidase plasmid (transfection control; Stratagene) and TurboFect *in vitro* transfection reagent (Fermentas). After 16 h, cells were exposed to 20 or 0.5% O_2_ for 2 h, as described above. Luciferase activity was determined using a Luciferase assay system (Promega) and normalized with respect to β-galactosidase activity, which was assessed using a β-galactosidase enzyme assay system (Promega) according to the manufacturer's instructions. Three independent transfections were performed in each case. For immunoprecipitation and immunoblotting analyses, cells were seeded into specially designed 25-cm^2^ glass flasks coated with 0.01% gelatin for one day, and then transfected with expression vectors by using the TurboFect *in vitro* transfection reagent, according to the manufacture's protocol.

### RNA isolation and quantitative PCR

Total RNA was extracted from RKO and MDA-MB-231 cells using the TRIzol reagent (Invitrogen) and treated with DNase I (New England Biolabs). Total RNA (1 mg) was used for cDNA synthesis with AccuPower RT PreMix (Bioneer, Daejeon, Republic of Korea), and the resulting cDNA was PCR amplified with the appropriate primer pairs: *HIF-1α*, 5′-CTG ACC CTG CAC TCA ATC AAG-3′ (forward) and 5′-TGG GAC TAT TAG GCT CAG GTG-3′ (reverse); *NQO1*, 5′-CCC TGC GAA CTT TCA GTA TCC-3′ (forward) and 5′-CTT TCA GAA TGG CAG GGA CTC-3′ (reverse); *18S rDNA*, 5′-CTG ACC CTG CAC TCA ATC AAG-3′ (forward) and 5′-TGG GAC TAT TAG GCT CAG GTG-3′ (reverse); *PDK1*, 5′-TTC ACG ATC TGG ACT CGA ACT-3′ (forward) and 5′-TTA TCC ACT GGG CCC ATC TTT-3′ (reverse); *PGK1*, 5′-ACC TTG CCT GTT GAC TTT GTC-3′ (forward) and 5′-GTG ACA GCC TCA GCA TAC TTC-3′ (reverse); *LDHA*, 5′-CAT CCT GGG CTA TTG GAC TCT-3′ (forward) and 5′-TGT CCC AAA ATG CAA GGA ACA-3′ (reverse) (Bioneer, Daejeon, Republic of Korea). The quantitative PCR was performed and analysed by CFX Connect Real-Time PCR Detection System (Bio-Rad).

### Immunoprecipitation and immunoblot analyses

For endogenous co-immunoprecipitation, cells were lysed using ice-cold RIPA/PBS (33% v/v) containing an inhibitor cocktail (Roche Applied Science), sodium orthovanadate and sodium fluoride. One milligram of total cell proteins were incubated with 25 μl of washed Protein G-magnetic beads (New England Biolabs) at 4 °C for 1 h. Cleared lysates were incubated for 5 h at 4 °C with 5 μg of mouse monoclonal HIF-1α, NQO1 or normal mouse IgG antibody. Then, the samples were incubated with 25 μl of washed Protein G-magnetic beads (New England Biolabs) at 4 °C for 1 h. The immunoprecipitation matrix-antibody complex was then washed three times with ice-cold RIPA/PBS (33% v/v) and the bound proteins were resolved by SDS–polyacrylamide gel electrophoresis and analysed by an immunoblot analysis. Signals were detected by enhanced chemiluminescence (Pierce). Uncropped images of the blots are shown in Supplementary Fig. 12.

### NQO1 enzyme activity

Cells were washed twice with phenol red-free Hank's balanced salt solution, resuspended in PBS (pH 7.2) containing 10 mg ml^−1^ aprotinin, sonicated four times using 10-second pulses on ice, and centrifuged at 14,000*g* for 20 min. The supernatants were collected, transferred into microcentrifuge tubes and stored at −80 °C until NQO1 enzyme activity could be assessed.

### Nuclear and cytoplasmic protein extraction

RKO cells exposed to 20 or 0.5% O_2_ for 2 h, and harvested at the indicated times. To obtain the cytoplasmic fraction, cells were resuspended in 300 μl of Tween 20 lysis buffer (25 mM Tris, pH 8.0, 0–50 mM NaCl, 2 mM EDTA, 1 mM phenylmethylsulphonyl fluoride, 0.5% Tween 20). The samples were incubated on ice for 15 min, and the cytoplasmic proteins were harvested by centrifugation at 6,000*g* for 1 min at 4 °C. The nuclear pellets were resuspended in 100 μl of Tween 20 lysis buffer containing 500 mM NaCl, incubated on ice for 15 min, and then mixed with 100 μl of NaCl-free Tween 20 lysis buffer. The nuclear proteins were harvested by centrifugation at 10,000*g* for 1 min at 4 °C. The extracted cytoplasmic and nuclear proteins (0.5 mg) were precipitated using 0.5 μg of anti-HIF-1α or anti-NQO1 antibody, and immunoprecipitation was performed as described above.

### Small-interfering RNA transfection

NQO1, PHD1, PHD2 and PHD3 were subject to RNA interference with 19-bp (including a 2-deoxynucleotide overhang) small-interfering RNA (siRNA). siRNAs against NQO1 (CCGUACACAGAUACCUUGAdTdT), PHD1 (GACAAGUAUCAGCUAGCAUdTdT), PHD2 (GAGUAGAGCAUAUAGAGAUdTdT) and PHD3 (CGUGUAUCGUUCCCUCUdTdT) were purchased from Bioneer Corporation (Daejeon, Republic of Korea). The StealthRNAi (Invitrogen) was used as a negative control. For transfection, cells were seeded in 25-cm^2^ flasks, grown to ∼80% confluence and transfected with siRNA duplexes using LipofectAMINE 2,000 (Invitrogen) according to the manufacturer's recommendations. After 48 h, the cells were processed as indicated for analysis.

### Ni-NTA and GFP bead-based pulldown assays

His_6_- and EGFP- tagged proteins were expressed in RKO or MDA-MB-231 cells. His_6_- and EGFP-tagged proteins were affinity-purified and subsequently conjugated to Ni-NTA resin (Qiagen) and anti-GFP beads (D153-10, MBL). His_6_-conjugated resin and EGFP-conjugated resin were incubated for 2 h at 4 °C with one milligram of total proteins of RKO cells exposed to hypoxia for 2 h. The resin and beads were extensively washed, eluted and analysed by an immunoblot analysis.

### Immunofluorescence and confocal microscopy

Slide-mounted cells were fixed with 3.7% paraformaldehyde and incubated in a blocking solution (3% BSA) for 1 h at room temperature. The slides were then incubated overnight at 4 °C with anti-NQO1 (NBP-1-31355, Novus) and anti-HIF-1α (sc-13515, Sata Cruz Biotechnology), as indicated. Thereafter, the cells were washed three times with blocking solution and then incubated with FITC- and Texas red- conjugated secondary antibodies for 1 h. The slides were washed twice with PBS and the cell nuclei were stained with 4,6-diamidino-2-phenylindole (Invitrogen) for 10 min. Coverslips were washed three times with PBS and mounted onto the slides using mounting reagent (Invitrogen) and the cells were analysed by laser-scanning confocal microscopy (TE2000-E; Nikon, Tokyo, Japan).

### Ubiquitination assays

Cells were exposed to 1% O_2_ for 2 h, and further incubated for 1 h in the presence or absence of 10 μg ml^−1^ MG132 (Calbiochem). After 1 h, the samples were harvested and lysed with lysis buffer containing N-ethylmaleimide. Supernatants were immunoprecipitated with an anti-HIF-1α antibody and the washed immunoprecipitates were resolved by SDS–polyacrylamide gel electrophoresis and probed with an anti-ubiquitin antibody.

### Animal studies

All mouse procedures in this study were approved by the Institutional Animal Care and Use Committee (IACUC) protocol approved for this study by Korea Institute of Radiological and Medical Sciences (KIRAMS 2013–70), Inha University (INHA 151,119–388) and the University of Texas at Dallas Institutional Animal Care and Use Committee (IACUC 13-04). Seven-week-old, female nude mice (BALB/c-nu) were purchased from Orient Bio Laboratory Animal Inc. (Seoul, Korea) and maintained at 25 °C with a 12-h light/12-h dark cycle under specific pathogen-free conditions with *ad libitum* access to sterile water and food. Mice were monitored daily for their health by the animal staff. Per IACUC guidelines mice were killed when their tumours size reached 2 cm diameter or their body weight decreased to 20% of the original body weight. To generate subcutaneous flank tumours, 2 × 10^4^ cells were resuspended in 100 μl of Hank's balanced salt solution and injected subcutaneously into the right flank of mice. Mice were assigned into 10–12 mice per group. Tumours were measured every day for 2 weeks and when tumours reached 150 mm^3^ in volume, with designated this as day 1. Tumour volume (TV) was calculated by using the following formula: TV=length × (width)^2^ × 0.5. Pimonidazole was used to generate hypoxic adducts by intraperitoneal injection at 60 mg kg^−1^ (Hydroxyprobe) 60 min before killing of mice.

### Immunohistochemistry

Paraffin-embedded sections were deparaffinized and rehydrated. HIF-1α or NQO1 immunohistochemistry was performed with a Vectastain Elite ABC kit (Vector Laboratories Inc.) following the manufacturer's protocol. For antigen retrieval, the sections were placed in citrate buffer (pH 6.0) and heated in a microwave oven for 10 min. For immunoperoxidase labelling, endogenous peroxidase was blocked by 0.3% H_2_O_2_ in absolute methanol for 15 min at room temperature. The sections were then incubated overnight at 4 °C with anti-CA9 (NB100-417, Novus) or anti-NQO1 and washed with PBS containing 0.05% Trion X-100. Incubation with secondary antibody and the peroxidase–antiperoxidase complex were carried out for 30 min at room temperature. Immunoreactive sites were visualized by 3,3′-DAB. Afterward, the slices were counterstained by haematoxylin. For the detection of NQO1 and HIF-1α co-localization, sections were incubated with rabbit anti-NQO1 and anti-HIF-1α overnight at 4 °C. Following washing, sections were incubated with anti-rabbit Alexa 488- and anti-mouse Alexa 546-conjugated secondary antibody (Molecular Probes) for 1 h at room temperature. Subsequently, sections were stained with 4,6-diamidino-2-phenylindole (1 μg ml^−1^) for 3 min. Hypoxia detection and pimonidazole staining was performed using a FITC conjugated primary antibody overnight at 4 °C.

### Statistical analysis

All grouped data are presented as mean ±s.d. Difference between groups was analysed by analysis of variance or Student's *t*-test using either SPSS or GraphPad Prism software. For the survival analysis, Kaplan–Meier curves were generated using either SPSS or Prism software and log-rank analysis were performed. All experiments were repeated in at least duplicate with triplicate technical replicates.

### Data availability

The TCGA, Ki, Skrzypczak, Kaiser, Staub, Reid and Smith data sets used in this study are available at http:www.oncomine.com. Data supporting the findings of this study are available within the article and its Supplementary Information files and from the corresponding author on reasonable request.

## Additional information

**How to cite this article:** Oh, E.-T. *et al*. NQO1 inhibits proteasome-mediated degradation of HIF-1α. *Nat. Commun.* **7,** 13593 doi: 10.1038/ncomms13593 (2016).

**Publisher's note:** Springer Nature remains neutral with regard to jurisdictional claims in published maps and institutional affiliations.

## Supplementary Material

Supplementary Information Supplementary Figures.
