# Supplementary material for: NQO1 inhibits proteasome-mediated degradation of HIF-1α
Source: Nat Commun. 2016 Dec 14;7:13593. doi: 10.1038/ncomms13593 (PMC5171868; doi:10.1038/ncomms13593)
Supplement: Supplementary Information — Supplementary Figures. [file ncomms13593-s1.pdf]

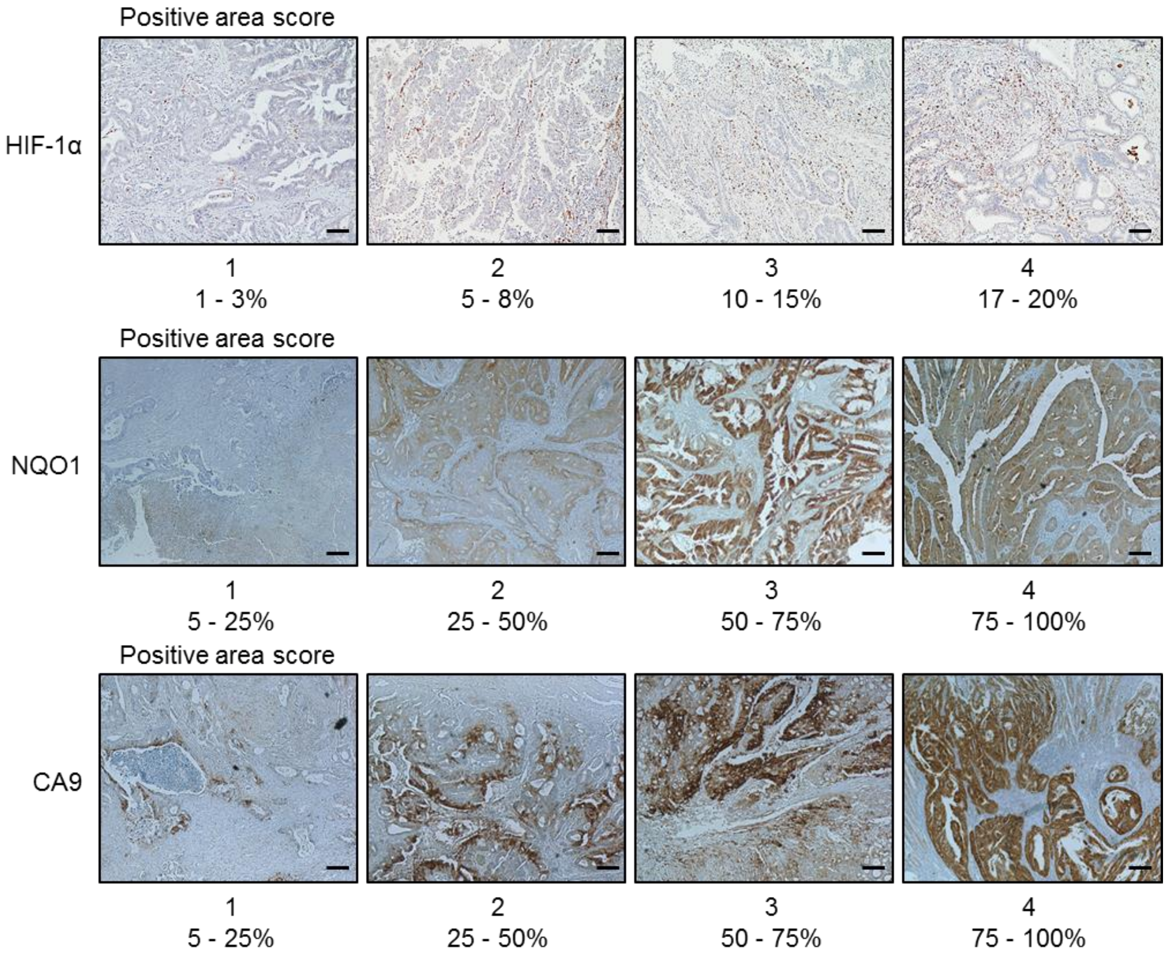

Supplementary Figure 1. Histopathological scoring criteria used for analysis of HIF-1α, NQO1 and CA9 staining in human clinical tumor tissue. HIF-1α IHC score was defined by positive area score (1-3%, 1; >5-8%, 2; >10-15%, 3; >17-20%, 4). IHC scores of NQO1 and CA9 were defined by positive area score (5-25%, 1; >25-50%, 2; >50-75%, 3; >75-100%, 4). Bar = 500 μm

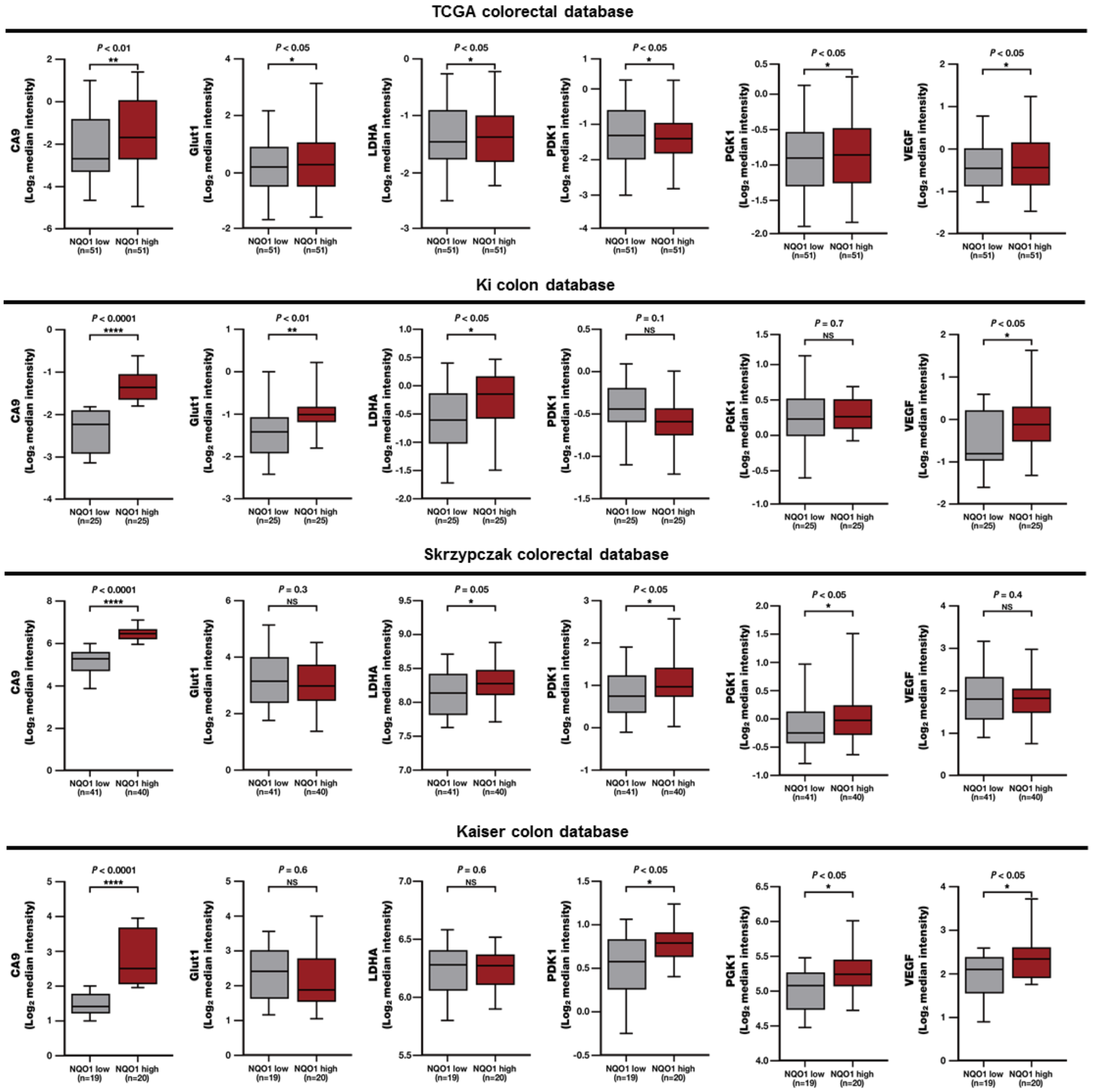

Supplementary Figure 2. Up-regulation of NQO1 correlates with expression of HIF-1α target genes in colorectal cancer patients. Oncomine analysis of the TCGA (n = 51 NQO1 high, n = 51 NQO1 low), Ki (n = 25 NQO1 high, n = 25 NQO1 low), Skrzypczak (n = 40 NQO1 high, n = 41 NQO1 low) and Kaiser (n = 20 NQO1 high, n = 19 NQO1 low) database indicates that the expression of NQO1 correlates with canonical HIF-1α target gene expression. For box plots, the center line represents median value, the box limits are at the 25th and 75th percentiles, and the whiskers represent minimum and maximum values. **P*<0.05 with unpaired *t*-test, ** *P* < 0.01 with unpaired *t*-test, **** *P* < 0.0001 with unpaired *t*-test.

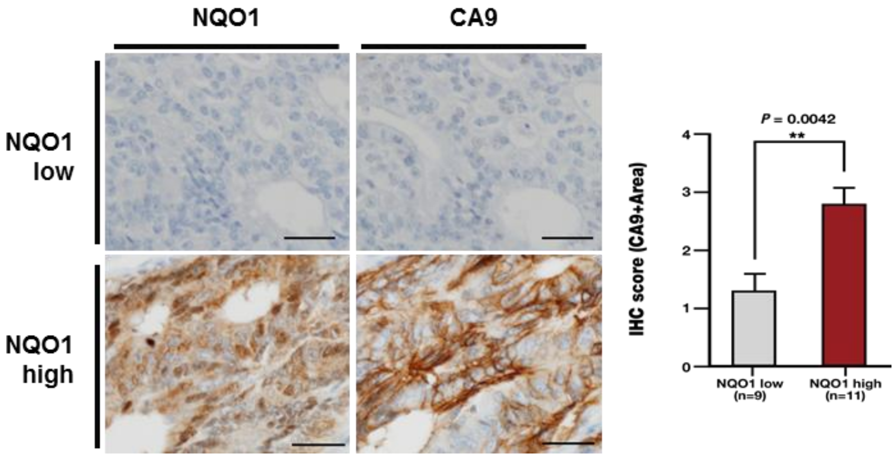

Supplementary Figure 3. Up-regulation of NQO1 correlates with expression of HIF-1α target gene, CA9, in colorectal cancer patients. Correlation between NQO1 and CA9 expression in colorectal cancer tissues. Immunohistochemical detection of CA9 in the high-level expression of NQO1 (n=11) compared to the low-level expression of NQO1 (n=9). Positive area score of CA9 was determined on the most characteristic areas. The positive area score of CA9 was evaluated by determining 10 high magnification power fields (×40). Statistical analysis of the average score of CA9 area score is shown in the right panel (P = 0.0218 with unpaired t-test). Bar = 50 μm

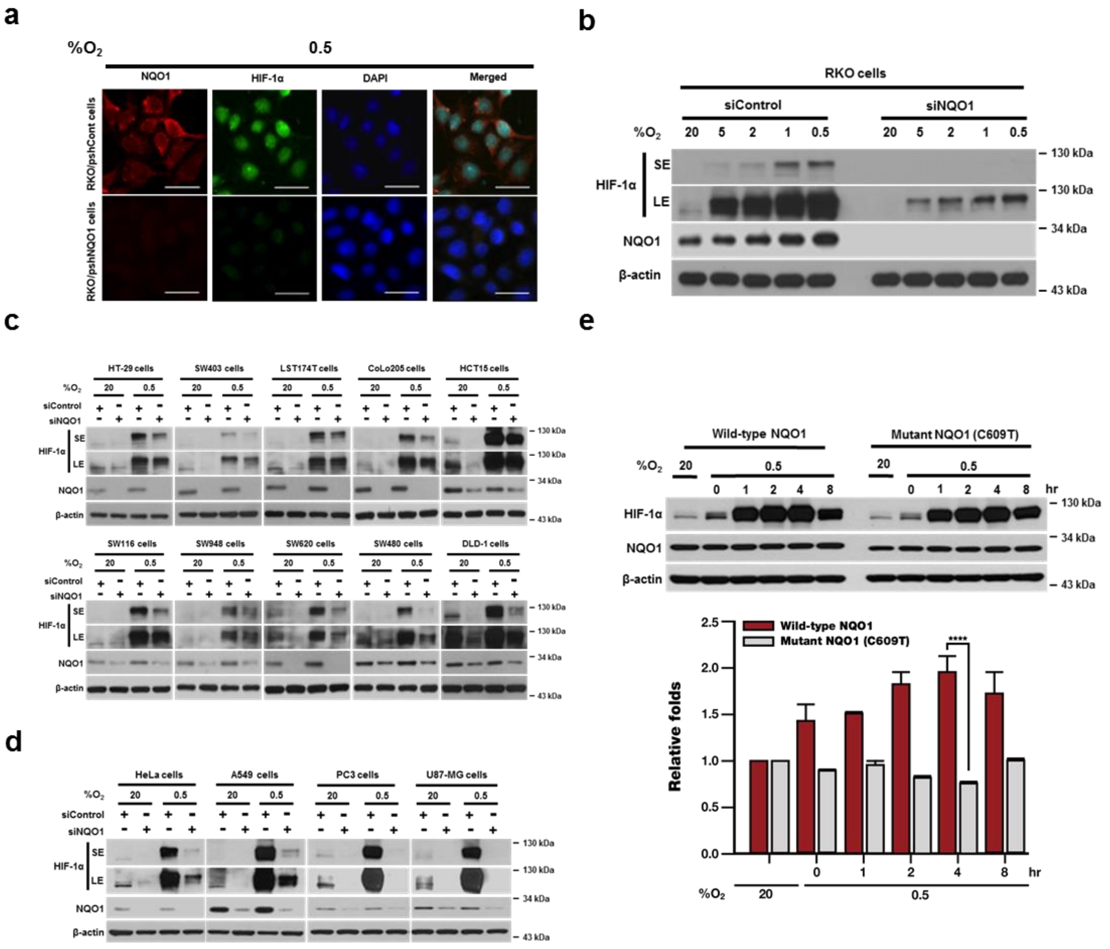

Supplementary Figure 4. Effect of NQO1 on transcription of *HIF-1α* and its stability. (a) NQO1 and HIF-1α expression levels in hypoxic RKO cells (0.5% O_2 for 2 hr) were assessed by immunocytochemical staining with anti-NQO1 primary and FITC-conjugated secondary antibodies, or anti-HIF-1α primary and Texas Red-conjugated secondary antibodies. DAPI was used to stain nuclei. **(b)** Effect of NQO1 on HIF-1α expression levels in various oxygen tensions; 0.5, 1, 2, 5 or 20% O_2. siControl- or siNQO1- transfected RKO cells were exposed to 0.5, 1, 2, 5 or 20% $O_2$for 2 hr and then harvested. Whole-cell lysates were analyzed by immunoblotting for HIF-1α, NQO1 and β-actin. SE, short exposure; LE, long exposure. **(c)** Human colorectal cancer cell lines, including RKO, HT-29, SW403, LST174T, CoLo205, HCT15, SW116, SW948, SW620, SW480, and DLD-1, were transfected with siNQO1. After 48 hr, the cells were exposed to 20% or 0.5% O_2 for 2 hr and then harvested. Whole-cell lysates were analyzed by immnoblotting for HIF-1α, NQO1 and β-actin. SE, short exposure; LE, long exposure. **(d)** A549, PC3, HeLa and U87MG cells were transfected with siNQO1. After 48 hr, the cells were exposed to 20% or 0.5% O_2 for 2 hr and then harvested. Whole-cell lysates were analyzed by immnoblotting for HIF-1α, NQO1 and β-actin. SE, short exposure; LE, long exposure. **(e)** MDA-MB-231/pNQO1 and MDA-MB-231/pNQO1 (C609T) cells were exposed to 20% or 0.5% O_2 for the indicated times. Whole-cell lysates were analyzed by immnoblotting for HIF-1α, NQO1 and β-actin (upper panel), and NQO1 reductase activity (lower panel). The results from three independent experiments are expressed as means ± SEM (P = 0.0001 with ANOVA).

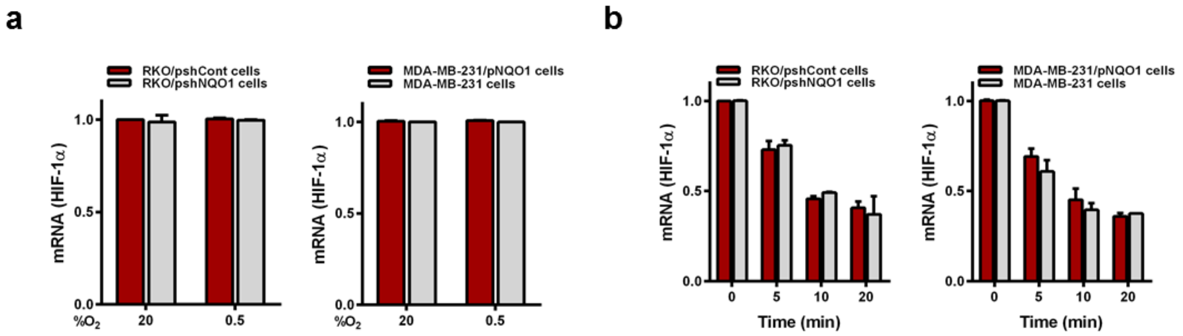

Supplementary Figure 5. NQO1 regulates HIF-1α stability in cancer cells. (a) HIF-1α mRNA expression is not affected by NQO1. RKO cells (Left) and MDA-MB-231 cells (Right) were exposed to 20% and 0.5% O_2 for 2 hr. qPCR was used to detect the expression levels of *HIF-1α*.The mRNA levels of HIF-1α and were examined by qPCR. *18S rRNA* was used as the internal control. **(b)** The effects of NQO1 onHIF-1α mRNA stability. RKO/pshCont and RKO/pshNQO1 cells were exposed to 20% (Left) or 0.5% (Right) O_2 for 2 hr, incubated with 5 μg/ml actinomycin D for 1 hr, and then harvested at the indicated times. The mRNA levels of HIF-1α and were examined by qPCR. *18S rRNA* was used as the internal control.

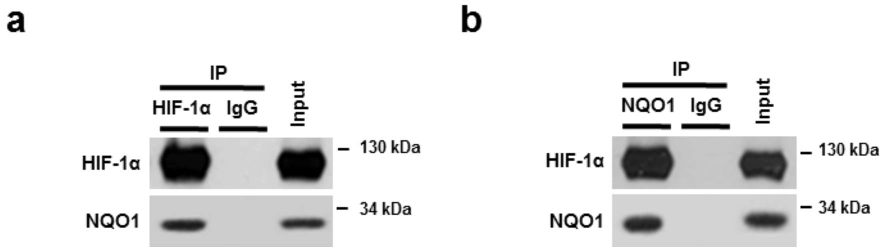

Supplementary Figure 6. NQO1 interacts with HIF-1α in the cytosol. (a,b) MEF cells were exposed to 0.5% O_2. After 2 hr, the cells were harvested and lysates were co-immunoprecipitated with anti-HIF-1α (a) or anti-NQO1 (b) antibody and anti-IgG antibody as a negative control. Then the precipitates were analyzed by an immunoblot analysis with anti -HIF-1α and -NQO1 antibodies.

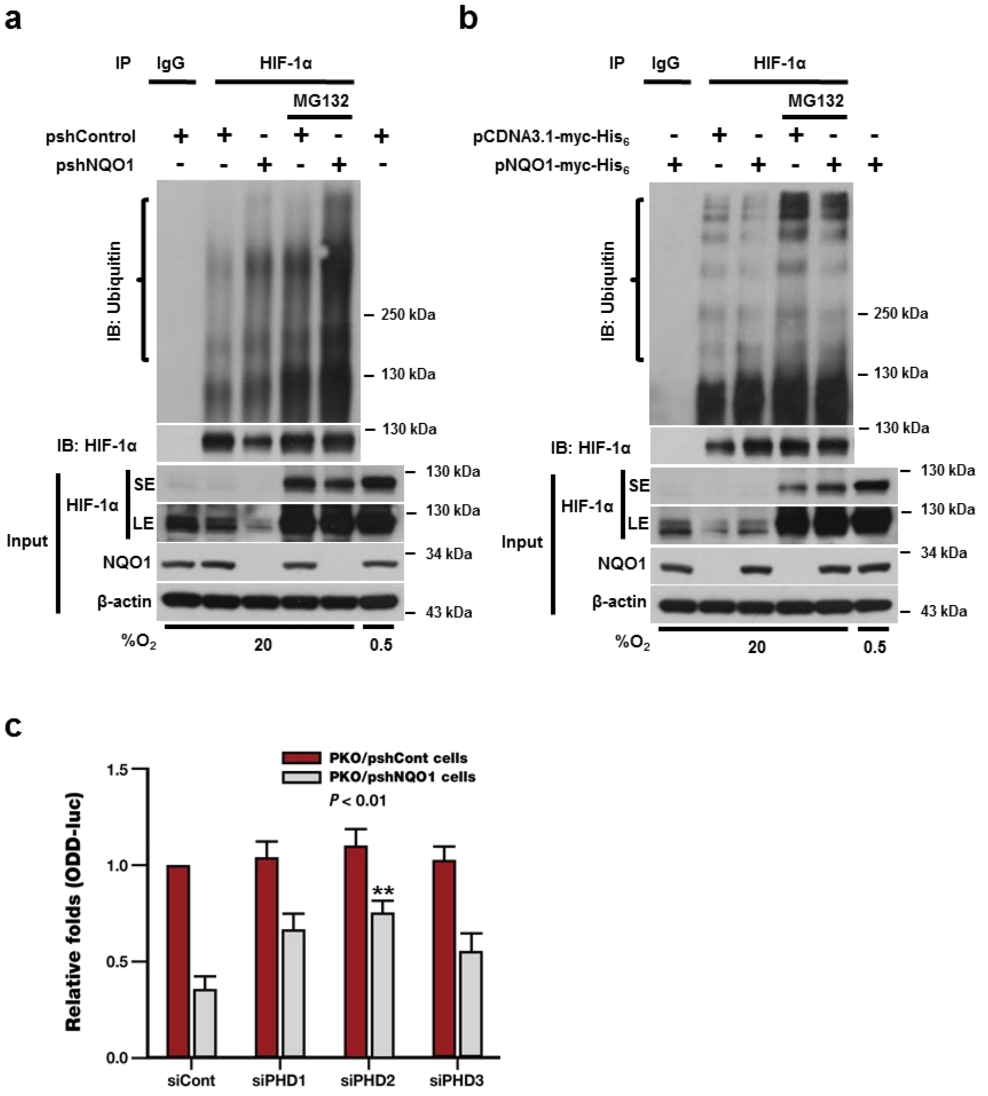

Supplementary Figure 7. NQO1 increases HIF-1α stability by inhibiting ubiquitination and proteasome-mediated degradation. (a,b) RKO (a) and MDA-MB-231 (b) and cells were transfected with pshControl or pshNQO1 and pCDNA3.1-myc-His$_6$ or pNQO1-myc-His$_6$ and, respectively. Under non-hypoxic conditions, cells were incubated for 1 hr in the presence or absence of MG132. Whole-cell extracts were immunoprecipitated with an anti- HIF-1α antibody, and ubiquitinated HIF-1α was detected with an anti-ubiquitin antibody. **(c)** RKO/shCont and RKO/pshNQO1 cells were transfected with siPHD1, siPHD2, or siPHD3. After 48 hr, the cells were co-transfected with p*ODD-luc* and pCMV-β-galactosidase for 16 hr, and then exposed to 0.5% O_2 for another 2 hr. Luciferase activity was normalized with respect to β-galactosidase activity (mean ± SD shown; ** P < 0.01 with ANOVA).

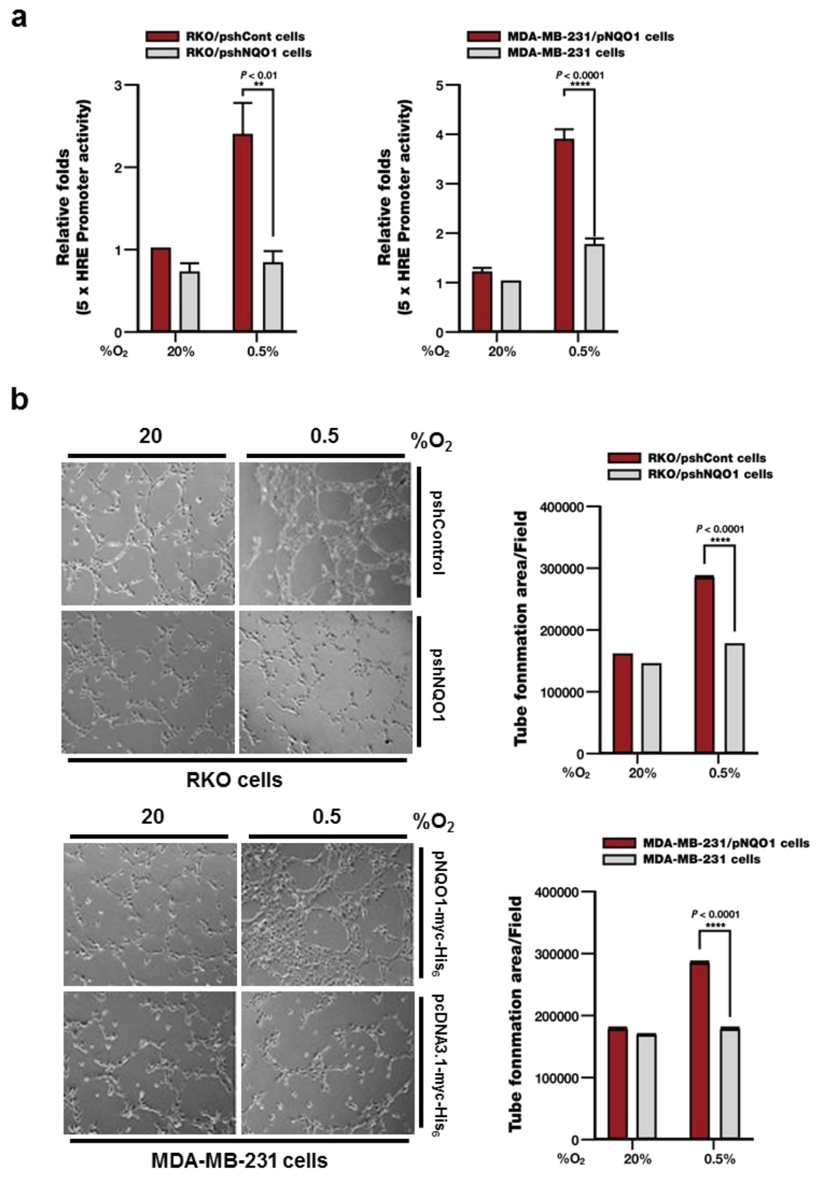

Supplementary Figure 8. NQO1 increases HIF-1α transcriptional activity. (a) RKO (Left) and MDA-MB-231 (Right) cells were transfected with pshCont or pshNQO1 and pCDNA3.1-myc-His$_6$ or pNQO1-myc-His$_6$, respectively, for 48 hr, further co-transfected with p5 × HRE-*luc* and pCMV-β-galactosidase for 16 hr, and then exposed to 20% or 0.5% O_2 for another 2 hr. Luciferase activity was normalized with respect to β-galactosidase activity (mean ± SD shown). ** P < 0.01 with ANOVA, **** P < 0.0001 with ANOVA. **(b)** RKO (upper panel) and MDA-MB-231 (lower panel) cells were exposed to 20% or 0.5% O_2 for 24 hr, and conditioned media were collected. HUVECs (1 × 10^5 per well) were seeded to 4-well plates in 200 μl of complete ECM plus 800 μl of conditioned media. After 6 hr of incubation, the area covered by the newly formed tubes was determined using the Image J program. The number of pixels in the tube area was quantified for three images per well at 40 × magnification (mean ± SD shown; **** P < 0.0001 with ANOVA).

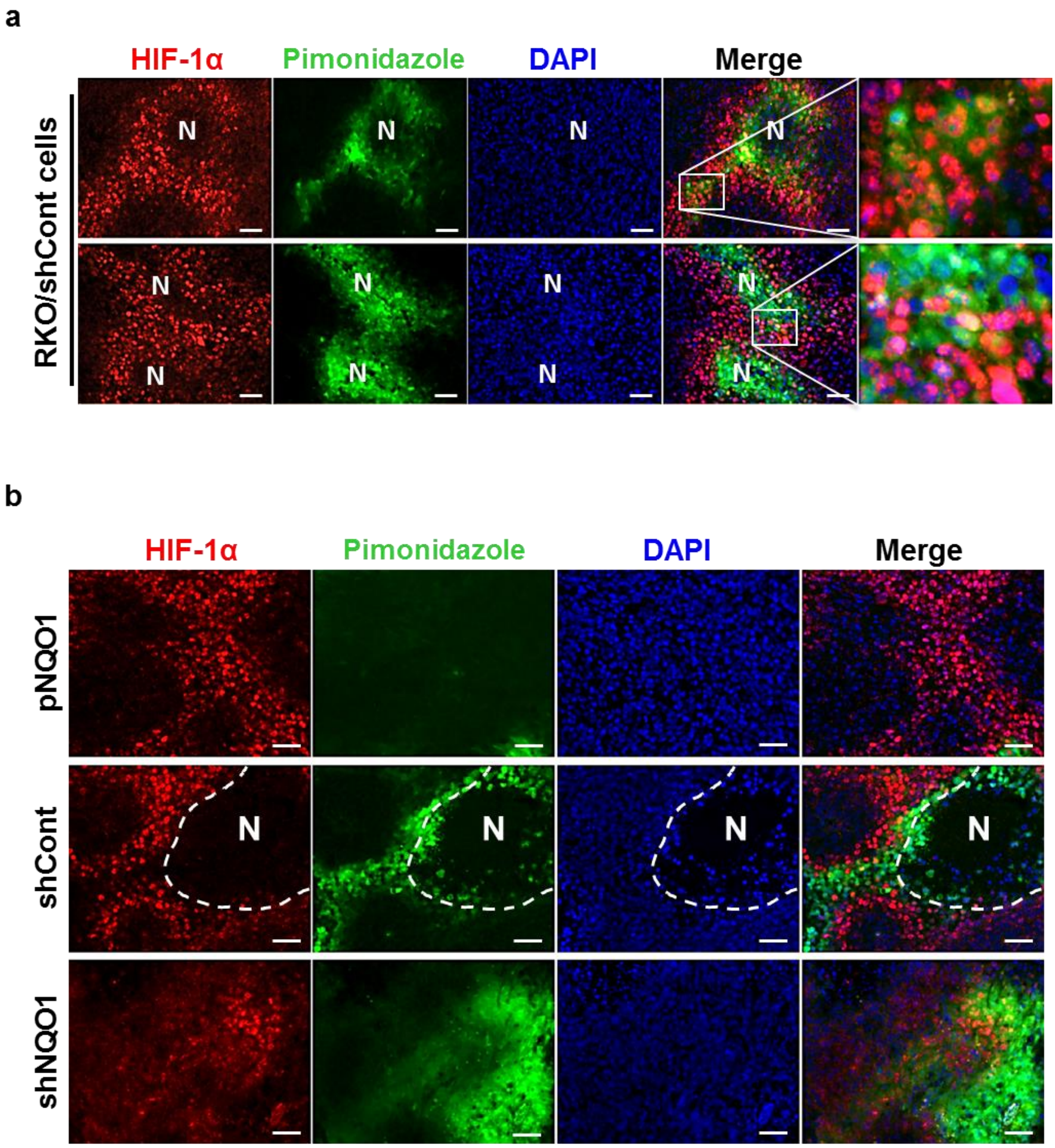

Supplementary Figure 9. NQO1 promotes HIF-1α stabilization. (a) RKO/shCont xenograft tumors were co-stained for pimonidazole (green) and HIF-1α (red). Scale bars = 100 μm. **(b)** HIF-1α expression and tissue hypoxia levels were assessed by immunohistochemical staining in RKO/pNQO1, RKO/pshCont, and RKO/pshNQO1 xenograft tumors. Hypoxic areas in tumors were detected by immunostaining for hypoxia probe, pimonidazole. N, necrotic areas; Scale bars = 100 μm.

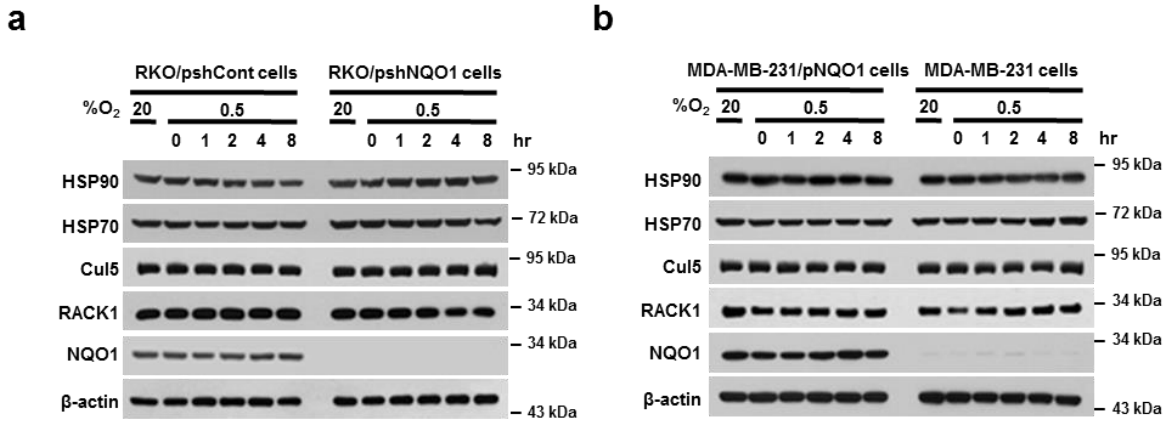

Supplementary Figure 10. Effects of NQO1 on expression of proteins related to HIF-1α stability. (a,b) Effects of NQO1 on the expression of proteins related to the degradation of HIF-1α. RKO cells (a) and MDA-MB-231 cells (b) were exposed to 20% and 0.5% O_2 for 8 hr, and cells were harvested at the indicated times. Whole-cell lysates were analyzed by immunoblotting for HSP90, HSP70, Cullin5, RACK1, NQO1, and β-actin.

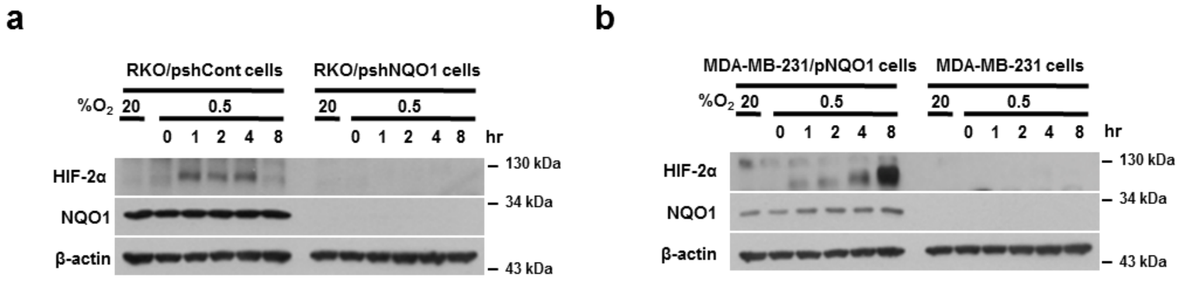

Supplementary Figure 11. NQO1 enhances HIF-2α expression. (a,b) Effects of NQO1 on the expression of HIF-2α. RKO cells (a) and MDA-MB-231 cells (b) were exposed to 20% and 0.5% O_2 for 8 hr, and cells were harvested at the indicated times. Whole-cell lysates were analyzed by immunoblotting for HIF-2α, NQO1, and β-actin.

Related to Fig. 2a

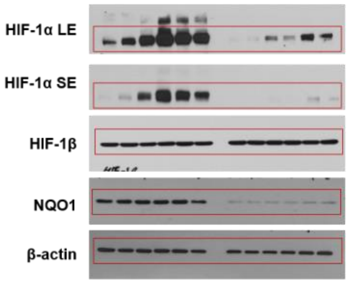

Related to Fig. 2b

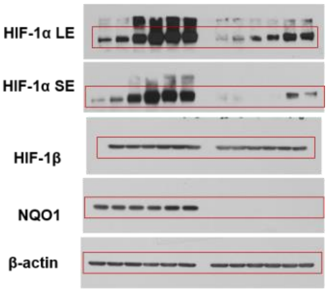

Related to Fig. 2c

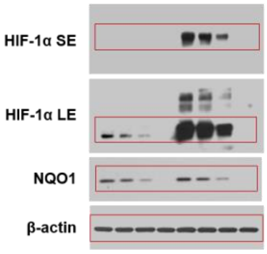

Related to Fig. 2d

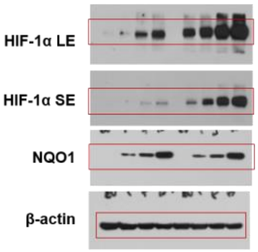

Related to Fig. 2e

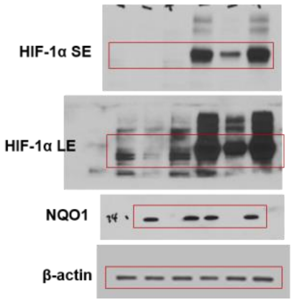

Related to Fig. 2f

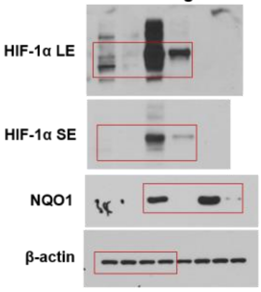

Related to Fig. 3a

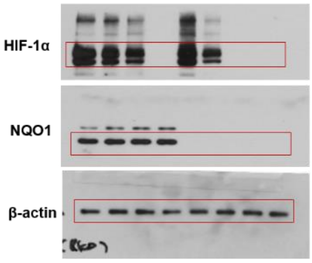

Related to Fig. 3b

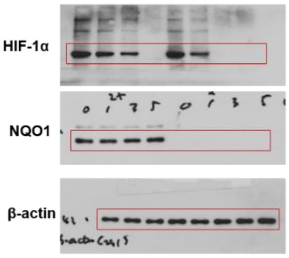

Related to Fig. 3c

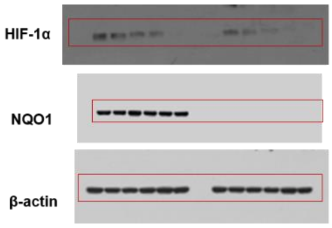

Related to Fig. 3d

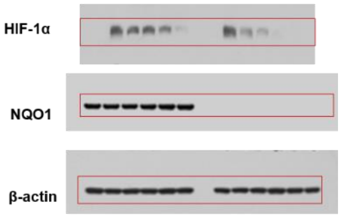

Related to Fig. 4a

HIF-1α

NQO1

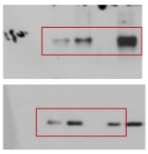

HIF-1α

NQO1

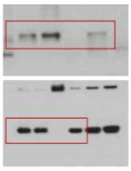

Related to Fig. 4b

HIF-1α

NQO1

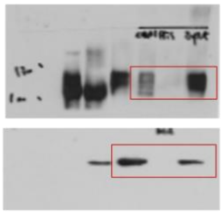

HIF-1α

NQO1

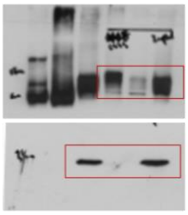

Related to Fig. 4d

HIF-1α

NQO1

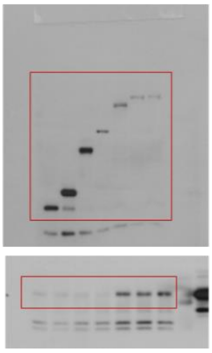

Related to Fig. 4e

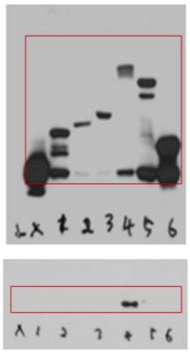

Related to Fig. 4f

HIF-1α

NQO1

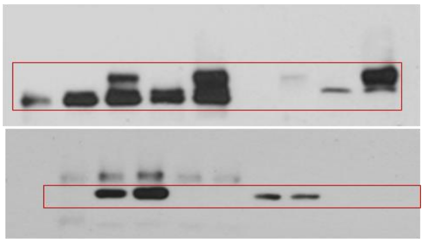

HIF-1α

NQO1

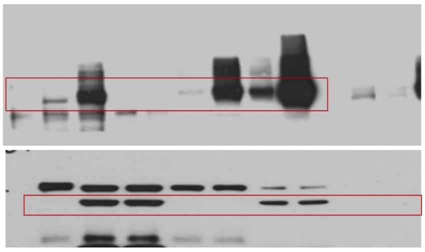

Related to Fig. 5a

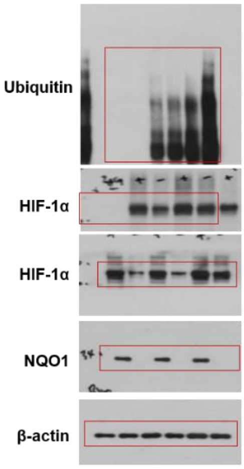

Related to Fig. 5b

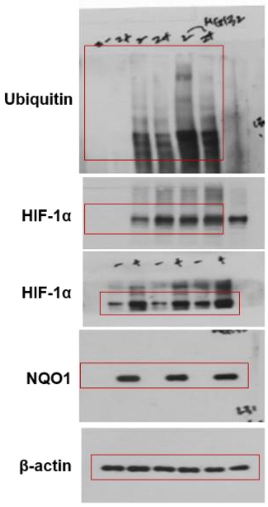

Related to Fig. 5c

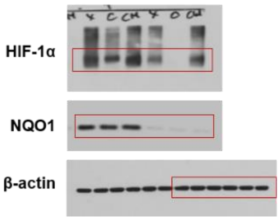

Related to Fig. 5d

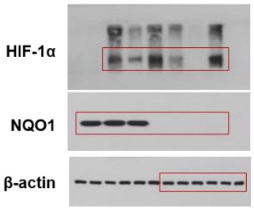

Related to Fig. 5e

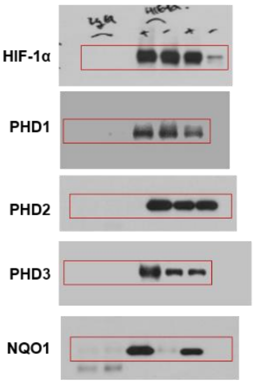

Related to Fig. 5f

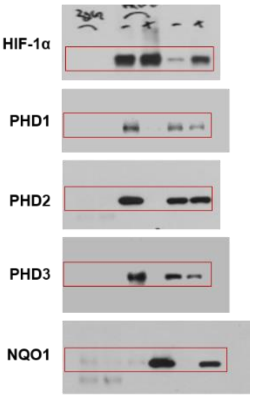

Related to Fig. 5g

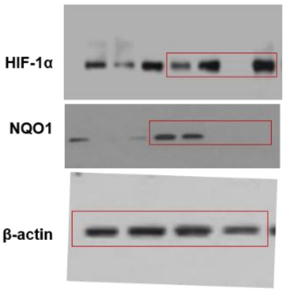

Related to Fig. 5h

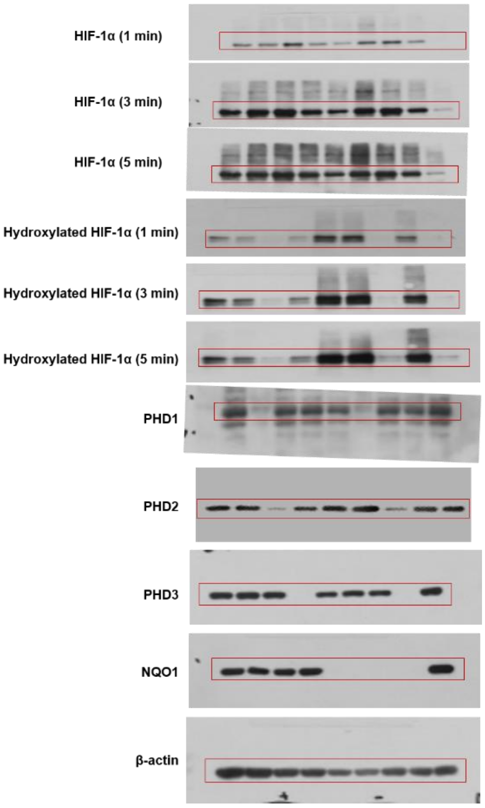

Related to Supplementary Fig. 4b

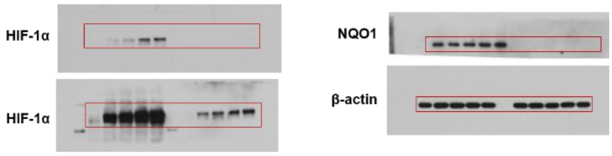

Related to Supplementary Fig. 4c

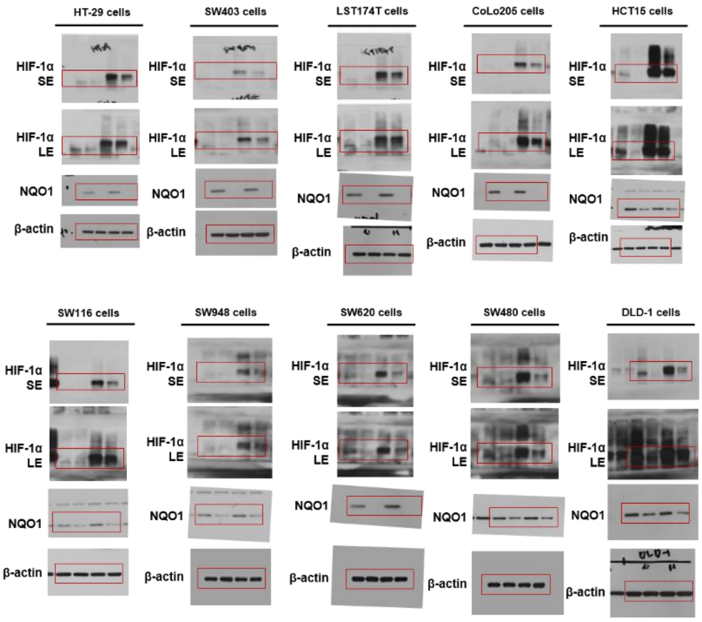

Related to Supplementary Fig. 4d

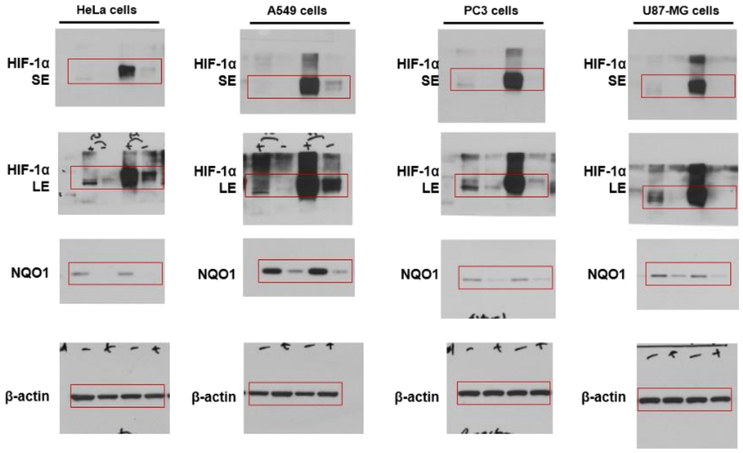

Related to Supplementary Fig. 4e

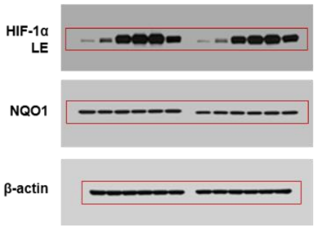

Related to Supplementary Fig. 6a

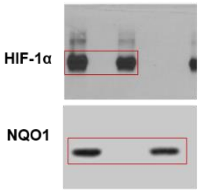

Related to Supplementary Fig. 6b

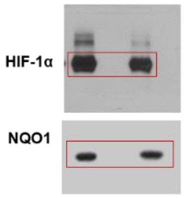

Related to Supplementary Fig. 7a

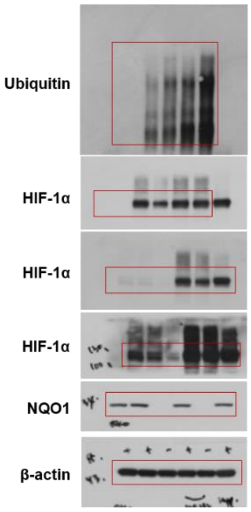

Related to Supplementary Fig. 7b

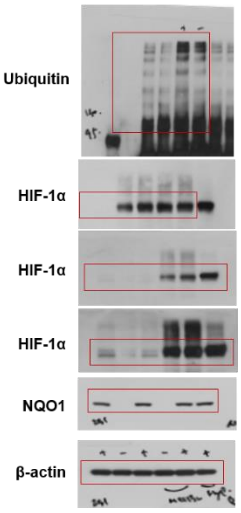

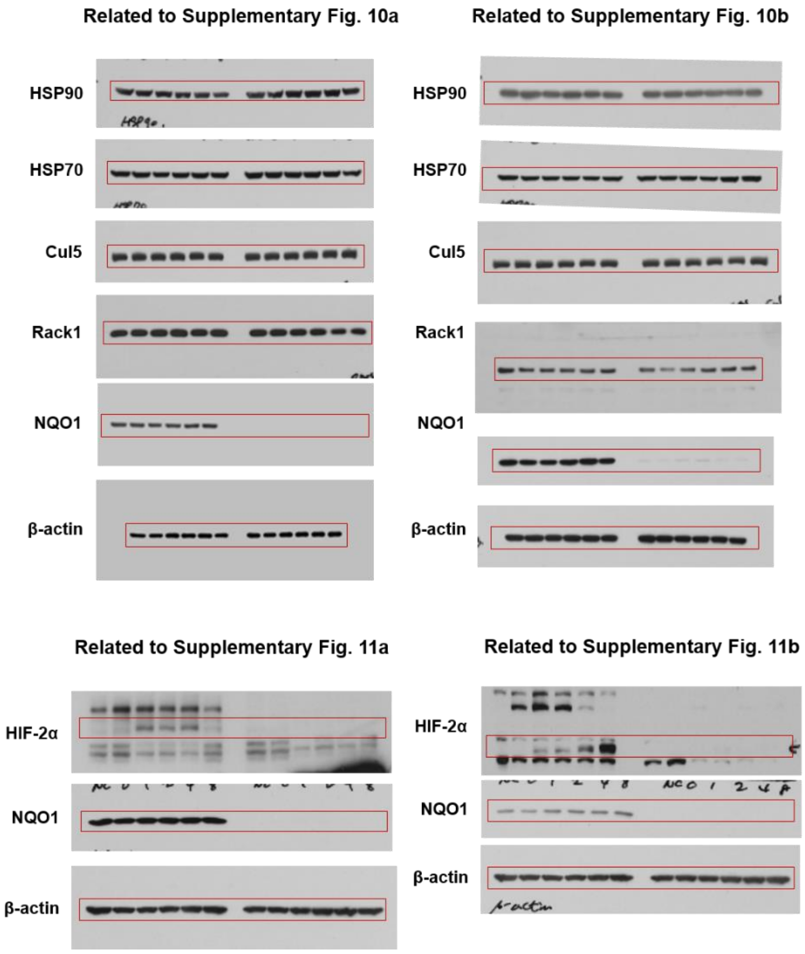

Supplementary Figure 12. Uncropped images of Immunoblots are presented. Reference to the original figures is presented for each blot. (LE = low exposreu; SE = short exposure).
